# Chitosan-Based Functional Films and Nanocomposites for Sustainable Electronics: A Structure–Property–Function Systematic Review

**DOI:** 10.3390/ijms27177942

**Published:** 2026-09-06

**Authors:** Muhammad Rif’an, Arie Aryanto, Karlisa Priandana, Waras Nurcholis

**Affiliations:** 1 Information Systems Study Program, Faculty of Science and Technology, Universitas Terbuka, Pamulang, South Tangerang 15418, Indonesia; 2 Biology Study Program, Faculty of Science and Technology, Universitas Terbuka, Pamulang, South Tangerang 15418, Indonesia; arie.aryanto@ecampus.ut.ac.id; 3 Computer Science Study Program, School of Data Science, Mathematics and Informatics, IPB University, Bogor 16680, Indonesia; karlisa@apps.ipb.ac.id; 4 Department of Biochemistry, Faculty of Mathematics and Natural Sciences, IPB University, Bogor 16680, Indonesia

**Keywords:** chitosan, functional films, sustainable electronics, dielectric nanocomposites, impedance sensing

## Abstract

Chitosan-based films and nanocomposites have attracted growing attention as renewable, biodegradable, and chemically adaptable materials for sustainable electronics. This systematic review synthesizes evidence on chitosan-containing functional films, membranes, polymer electrolytes, dielectric substrates, optoelectronic nanocomposites, electrochemical sensors, impedance sensors, and smart-device interfaces. This review adopts a structure–property–function perspective, examining molecular interactions, crystallinity, amorphous fraction, morphology, filler dispersion, processing routes, and interfacial architecture in relation to optical, electrical, dielectric, electrochemical, and sensing performance. The literature was identified using Scopus-oriented Boolean searches combining chitosan/chitin terms with thin-film or membrane descriptors, electronic-function terms, and structure–property terminology. Relevant studies were grouped into four themes: structural engineering and processing, dielectric/electrical/impedance properties, optoelectronic and band-gap engineering, and electrochemical/impedance-sensing applications. The synthesis shows that chitosan becomes electronically functional when its semi-crystalline, hydrogen-bonded matrix is modified through salt doping, plasticization, blending, conductive polymers, carbon materials, metal oxides, metal–organic frameworks, or noble metal nanoparticles. Reported advances include ionic conductivities up to 10^−3^ S/cm, improved dielectric behavior, band-gap reduction, and low detection limits. However, inconsistent reporting of material source, molecular weight, degree of deacetylation, film thickness, humidity, stability, and sustainability metrics limits comparability.

## 1. Introduction

The search for sustainable electronic materials has become a central concern in polymer science and device engineering. Many conventional electronic materials still depend on petroleum-derived polymers, non-degradable substrates, high-temperature processing, or inorganic components that raise toxicity and end-of-life concerns. In response, research has increasingly turned to renewable, biodegradable, and low-toxicity materials that can support flexible electronics, sensors, energy-storage devices, biointerfaces, and smart packaging systems. Chitosan occupies a distinctive position in this transition because it combines natural abundance, biodegradability, film-forming ability, chemical functionality, and compatibility with a broad range of organic and inorganic modifiers. Reviews of biopolymer-based bioelectronics have emphasized that chitosan and related natural polymers can support soft, flexible, stimulus-responsive, and biologically compatible platforms for e-skin, implantable interfaces, biosignal detection, robotics, and next-generation bioelectronic devices [1]. Chitosan-based materials have also been described as versatile chemosensing platforms because their amine and hydroxyl groups can interact with metal ions, anions, biomolecules, and environmental analytes [2,3].

Chitosan should therefore not be viewed only as passive biodegradable support. It can serve as a functional matrix for membranes, solid polymer electrolytes, conductive films, dielectric substrates, optoelectronic composites, and sensor interfaces. Biopolymer electrolyte membranes containing chitosan, cellulose, and starch have been explored as alternatives to less environmentally favorable proton-exchange materials in sustainable primary redox batteries, producing open-circuit voltages above 0.75 V and power outputs up to 2.5 mW cm^−2^ [4]. Related polysaccharide-based systems have also been developed for flexible zinc-ion hybrid supercapacitors and eco-friendly conductive supercapacitor materials [5,6]. Chitosan-based substrates have recently been reported for flexible, printable, and sustainable organic electronic devices, while doped poly(3-hexylthiophene) coatings on chitosan have offered a direct route toward bio-based flexible electronics [7,8]. Taken together, these studies show that chitosan can move beyond the role of a bio-coating and operate as an electronically active platform.

The growth of chitosan-based electronics is evident across several research streams. In solid polymer electrolytes, chitosan blends can host ammonium, lithium, potassium, magnesium, and silver-ion systems, and their conductivity can be adjusted through salt dissociation, plasticization, and amorphous-phase formation. For example, chitosan:dextran electrolytes doped with NH_4_CF_3_SO_3_ reached a DC conductivity of 6.24 × 10^−6^ S/cm and displayed dielectric and impedance behavior relevant to EDLCs, fuel cells, sensors, actuators, and solar-cell-related devices [9]. Plasticized silver-ion-conducting chitosan-based nanocomposites showed a conductivity of 3.7 × 10^−4^ S/cm and electrochemical stability up to 2.1 V, while chitosan:potato starch systems plasticized with glycerol reached 1.62 × 10^−3^ S/cm with an ionic transference number of 0.989 [10,11]. These findings indicate that the electrical performance of chitosan is highly dependent on structure and processing rather than being determined solely by its native polymeric state.

In optoelectronic and dielectric films, chitosan is increasingly engineered through nanofiller incorporation, metal-oxide doping, MOF integration, and polymer blending. Chitosan/carboxymethyl cellulose films containing ZnO and Al_2_O_3_ nanofillers showed band-gap narrowing, dielectric enhancement, and mechanical improvement relevant to dielectric capacitors and optoelectronic substrates [12]. ZrO_2_-reinforced chitosan nanocomposites narrowed the optical band gap from 5.4 to 2.9 eV and increased the dielectric constant [13], while α-Fe_2_O_3_-loaded PVA/chitosan films showed band-gap reduction together with increased dielectric constant and AC conductivity [14]. Rare-earth and metal-oxide-co-doped chitosan films similarly exhibited reduced crystallite size, improved thermal behavior, narrowed optical gaps, and enhanced dielectric response [15]. MOF-doped chitosan systems provide especially strong evidence of electronic tunability: FeMOF-doped chitosan reduced the direct band gap from 5.5 to 2.0 eV, and CrMOF/chitosan films reduced the band gap to approximately 3 eV while increasing Urbach energy [16,17].

Sensor-oriented studies further show that chitosan can support interface engineering, immobilization, molecular recognition, and impedance modulation. Chitosan–polyaniline bio-nanocomposites on microelectrodes functioned as chemiresistive and chemicapacitive elements, with device behavior governed by morphology, conductive-network formation, and composition [18]. ZnO/CdWO_4_/chitosan flexible mixed-oxide films produced emissions relevant to optical devices and intelligent textile-based sensor platforms [19]. Collectively, these examples reveal a rich but fragmented literature: energy-device studies foreground ionic transport, optoelectronic studies emphasize band-gap and dielectric tuning, and sensor papers focus on sensitivity, detection limit, selectivity, response time, and real-sample testing. The central challenge is that these findings are rarely brought together within a systematic structure–property–function framework.

This review addresses that challenge through four objectives. First, it maps the main classes of chitosan-based functional films and nanocomposites used in sustainable electronics. Second, it synthesizes how structural parameters and processing routes influence dielectric, optoelectronic, electrochemical, and impedance properties. Third, it compares device-level functions across energy, sensing, bioelectronic, and smart-device applications. Fourth, it identifies methodological limitations, reporting gaps, and future research directions for scalable green electronic materials. The contribution of this review is therefore not a catalogue of chitosan applications but a framework explaining why and how chitosan-based materials become functional electronic materials when molecular structure, film architecture, charge-transport pathways, and device function are intentionally co-designed.

## 2. Methods

This systematic review followed the PRISMA 2020 statement and adapted Cochrane principles of explicit searching, transparent selection, reproducible extraction, and critical appraisal to materials- and device-level evidence, since a thematic synthesis approach was more appropriate than meta-analysis for this heterogeneous evidence base [20,21,22,23,24]. The review protocol was not prospectively registered.

### 2.1. Search Strategy and Eligibility Criteria

Records were identified in Scopus (17–26 April 2026) using TITLE-ABS-KEY ((chitosan OR chitin) AND (“thin film*” OR membrane* OR coating* OR biopolymer*) AND (electronic* OR dielectric* OR optoelectronic* OR sensor*)), built from five conceptual blocks (material; structure/nanocomposite; property/performance; function/application; processing/modification) combined with OR within and AND across blocks and restricted to 2015–2026, article or review type, English language, and journal source. Supplementary Table S1 reports four additional scoping queries (green-electronics-, structure–property-, waste-origin-, and impedance/optoelectronic-focused) used to position the review’s scope, together with the full keyword blocks; Supplementary Figure S1 shows the Boolean search strategy map.

Studies were eligible when they investigated chitosan, chitin, or chitosan-containing blends, films, membranes, coatings, polymer electrolytes, nanocomposites, or sensor interfaces and reported at least one measurable electronic, optical, dielectric, impedance, electrochemical, or sensing property in peer-reviewed journal articles or reviews indexed in Scopus. Studies addressing only food preservation, drug delivery, wound healing, antimicrobial activity, or biomedical scaffolds were excluded unless they also reported device-relevant evidence, as were duplicates, non-English documents, editorials, patents, inaccessible full texts, and records lacking sufficient structural, property, or performance data.

### 2.2. Screening, Selection, and Quality Assessment

**Figure 1:**
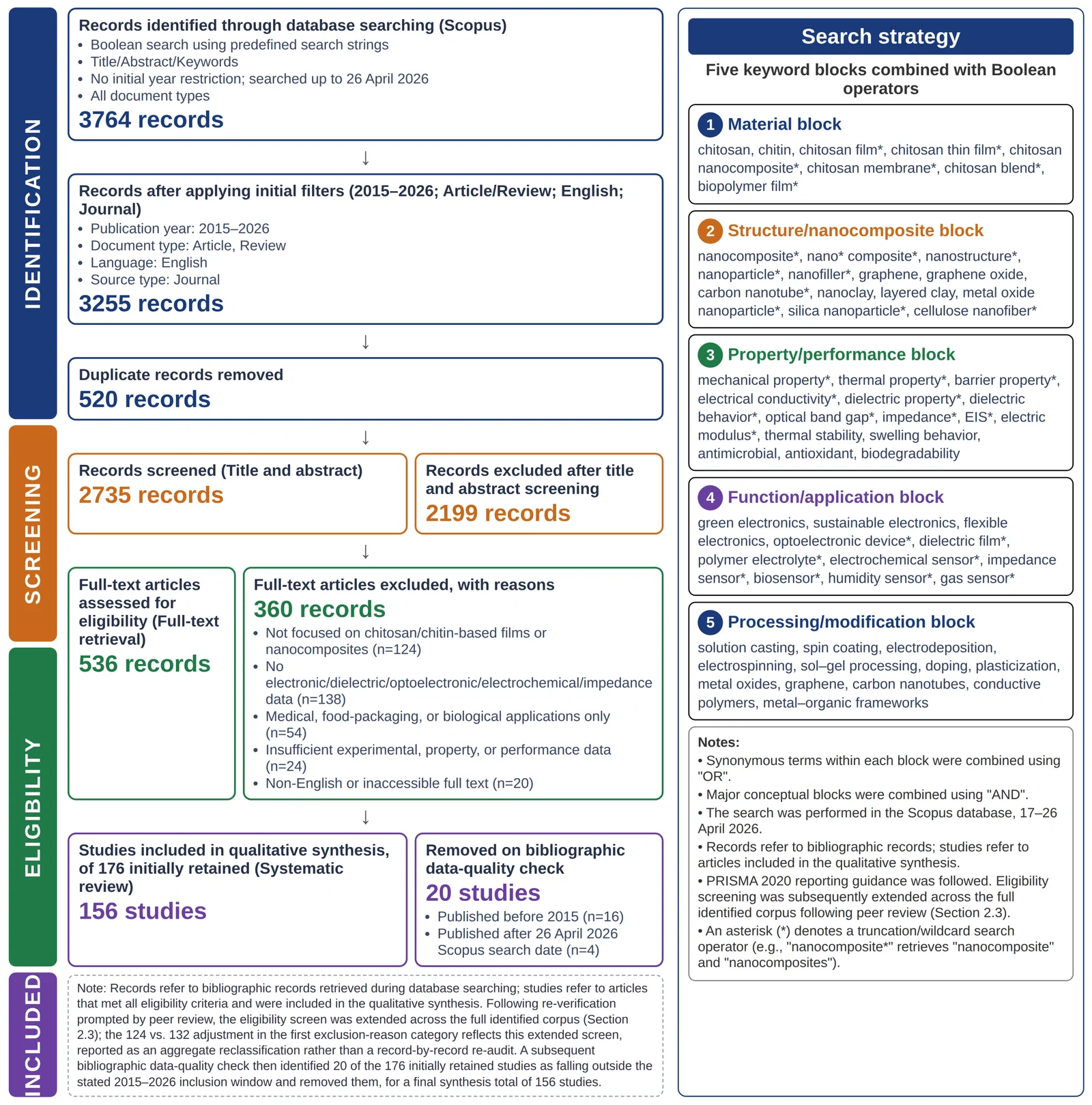
PRISMA flow diagram showing literature identification, initial filtering, duplicate removal, title and abstract screening, full-text eligibility assessment, reasons for exclusion, and final inclusion in the qualitative synthesis.

Screening followed the PRISMA logic of identification, duplicate removal, title/abstract screening, full-text eligibility assessment, and final inclusion (Figure 1).

The main search identified 3764 records; after year, document-type, language, and source filters, 3255 remained; duplicate removal excluded 520; title/abstract screening excluded 2199 of the remaining 2735; full texts were assessed for 536 records, of which 360 were excluded with documented reasons (not focused on chitosan/chitin-based films or nanocomposites, *n* = 124; no electronic/dielectric/optoelectronic/electrochemical/impedance data, *n* = 138; medical, food-packaging, or biological applications only, *n* = 54; insufficient experimental, property, or performance data, *n* = 24; non-English or inaccessible full text, *n* = 20). The full-text screening process initially retained 176 studies. A subsequent bibliographic data-quality check identified 20 of these as falling outside the stated inclusion window—16 published before 2015 and 4 published after the 26 April 2026 Scopus search date—and these were removed; the final qualitative synthesis therefore comprises **156 studies**, organized into four thematic groups: structural engineering and processing routes, dielectric/electrical/impedance properties, optoelectronic and band-gap engineering, and electrochemical/impedance-sensing applications.

Methodological quality was assessed against the clarity of material composition and synthesis procedure, reporting of chitosan source and concentration, adequacy of structural and functional characterization, measurement reliability, reproducibility indicators, and appropriateness of sustainability claims, with particular attention to consistent reporting and interpretation of impedance spectroscopy, Nyquist/Bode analysis, equivalent-circuit fitting, dielectric constant, dielectric loss, and electric modulus for relevant studies [25,26,27], including distinguishing chemiresistive, chemicapacitive, and electrolyte behaviors via modified Randles circuits [18].

### 2.3. Data Extraction and Use of Generative Artificial Intelligence

A structured extraction matrix recorded bibliographic information, chitosan form, fabrication method, dopant/filler/plasticizer, structural characterization, optical/electrical/dielectric/impedance properties, target device, performance metrics, sustainability claims, and limitations, prioritizing quantitative data (conductivity, dielectric constant, band gap, refractive index, ionic transference number, electrochemical stability, response/recovery time, limit of detection, linear range, sensitivity, stability) where available.

**Figure 2:**
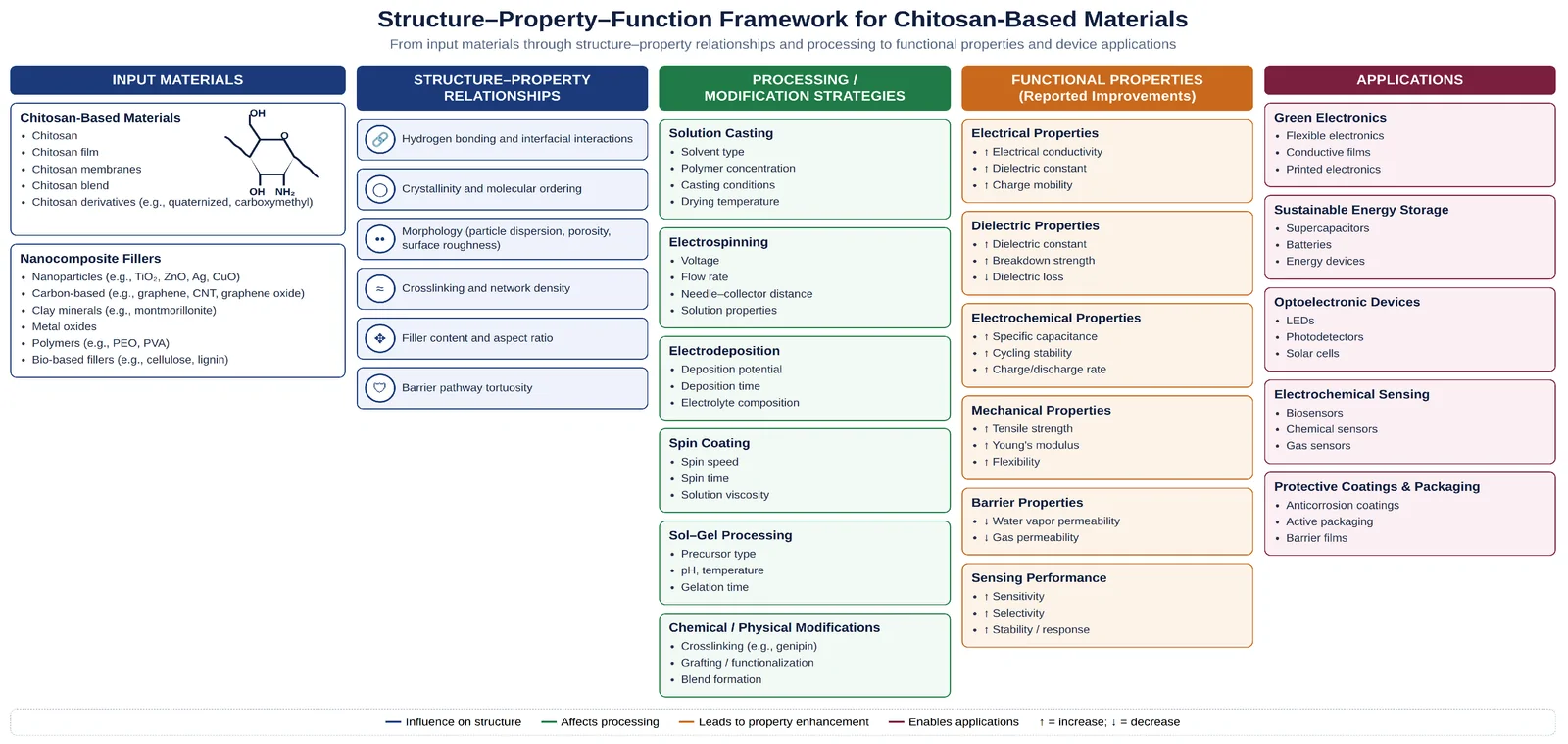
Structure–property–function framework summarizing how material composition, processing strategies, and structural features collectively determine the functional performance and application outcomes of chitosan-based films and nanocomposites.

**Figure 3:**
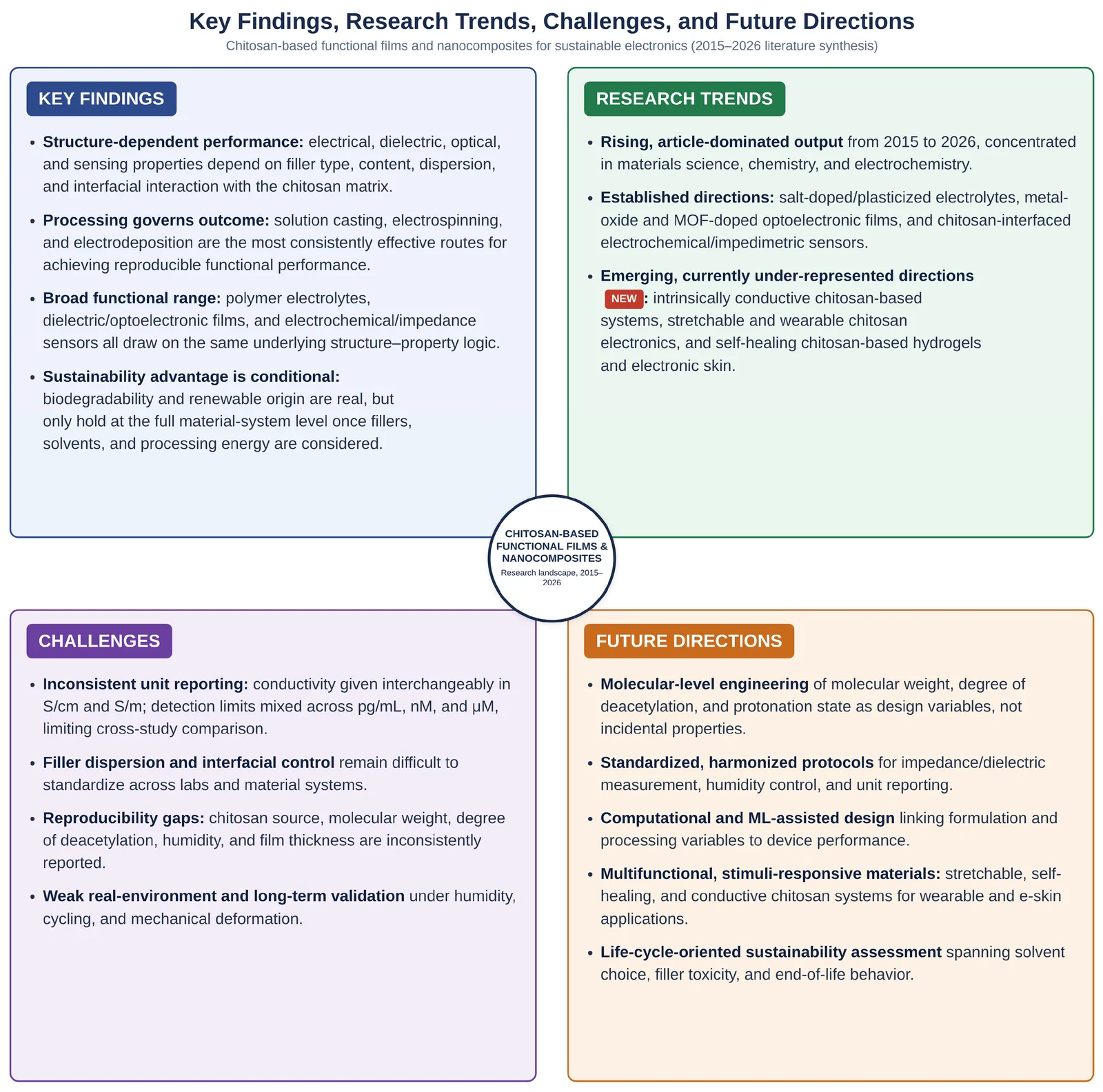
Key findings, research trends, challenges, and future directions in chitosan-based functional films and nanocomposites, consolidating the emerging-topics analysis below with the design trade-off discussion above.

**Figure 4:**
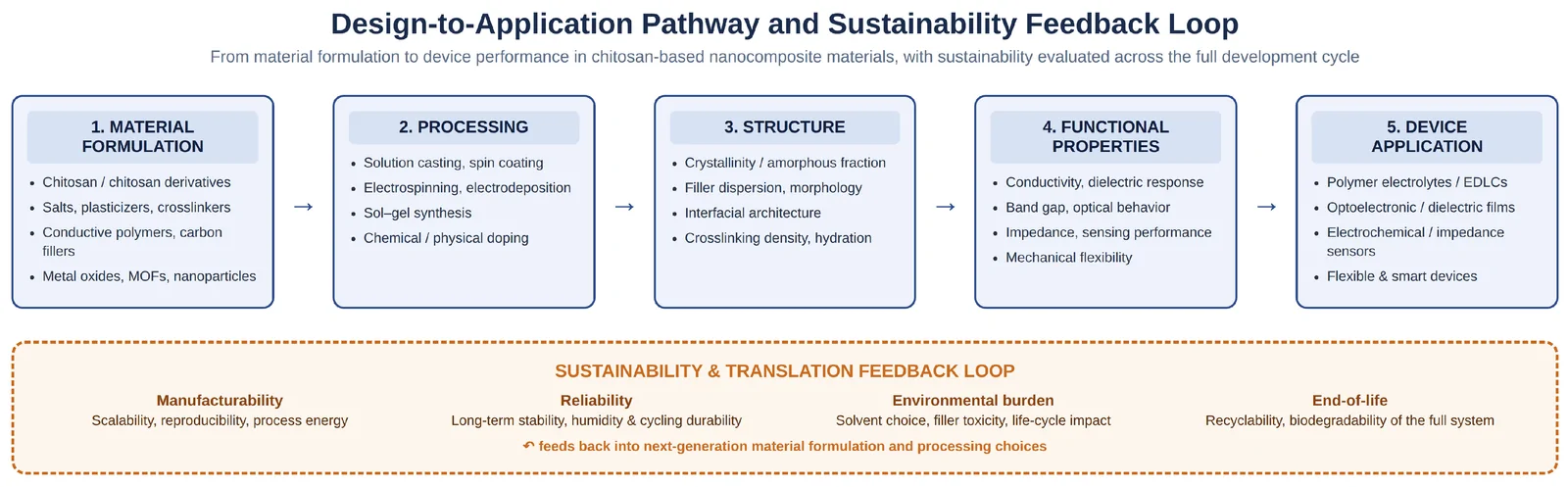
Design-to-application pathway for chitosan-based nanocomposite materials, coupled with the sustainability and translation feedback loop that links device performance back to material and process choices.

During preparation of this review, the authors used ChatGPT 5.5 (OpenAI) to assist with manuscript drafting, language refinement, organization of synthesized evidence, and preparation of graphical illustrations (Figure 1, Figure 2, Figure 3 and Figure 4) and captions. All AI-assisted outputs were critically reviewed, verified against the cited literature, and revised and approved by the authors, who determined the search strategy, study selection, evidence synthesis, interpretation, and conclusions and take full responsibility for the accuracy, originality, and integrity of the work.

## 3. Results and Discussion

Following the PRISMA-based selection process, the final qualitative synthesis included 156 studies that met the eligibility criteria and reported structural, electrical, dielectric, optoelectronic, electrochemical, impedance, or sensing evidence relevant to chitosan-based functional films and nanocomposites. The studies included were organized into four thematic groups: structural engineering and processing routes, dielectric/electrical/impedance properties, optoelectronic and band-gap engineering, and electrochemical/impedance-sensing applications. This thematic organization reflects the structure–property–function framework adopted in this review and enables comparison across material design, processing strategy, functional performance, and device relevance.

### 3.1. Molecular and Structural Basis of Chitosan as a Functional Electronic Biopolymer

The relevance of chitosan for electronics originates from its molecular architecture. Chitosan contains amino and hydroxyl groups that participate in hydrogen bonding, protonation, ionic complexation, metal coordination, and interfacial interactions. These interactions govern film formation, crystallinity, amorphous content, swelling behavior, filler compatibility, and charge mobility. Molecular modeling and blending studies have shown that chitosan combined with gelatin or starch can alter band-gap energy and dipole moment through hydrogen-bond-mediated structural changes [28]. These findings support the view that chitosan is not electronically inert; instead, its chemical structure can be tuned to generate charge-transport and polarization behavior.

Electronic functionality becomes more apparent when chitosan is positioned at interfaces or blended with conductive or ionic components. In a Au/CTS/n-InP hybrid junction, a chitosan interfacial layer increased the barrier height and reduced reverse-bias leakage current by approximately three orders of magnitude, indicating that chitosan can passivate semiconductor junctions and modify charge injection [29]. In polymer electrolyte systems, chitosan:methylcellulose films doped with LiI showed conductivity behavior consistent with Jonscher’s power law, correlated barrier hopping, Arrhenius-type conduction, and non-Debye relaxation [30]. PVA/chitosan solid polymer electrolytes loaded with NaTiO_3_ and rare-earth ions also showed reduced crystallinity, band-gap narrowing, improved dielectric response, and increased AC conductivity, supporting the interpretation that salt dissociation and nanofiller incorporation create electronically functional pathways [31].

### 3.2. Evolution from Passive Biopolymer Coatings to Active Green Electronic Materials

Historically, chitosan was often introduced as a film-forming, antimicrobial, edible, or biocompatible coating. In sustainable electronics, however, its role has shifted toward that of an active matrix. Chitosan can immobilize enzymes and recognition elements, host mobile ions, stabilize nanoparticles, form conductive polymer networks, regulate morphology, and support flexible substrates. PEG/chitosan–AgNO_3_ nanocomposites illustrate this transition: silver nitrate incorporation decreased the optical energy gap and increased the dielectric constant, dielectric loss, and conductivity, suggesting that localized states and increased amorphous character contributed to electronic tuning [32]. Nb_2_O_5_-doped chitosan/polyaniline systems and grafted chitosan–PANI networks further demonstrate how conductive polymers and oxide nanoparticles can convert chitosan into flexible electronic materials [33,34].

Nanoscale filler architecture is central to this evolution. Chitosan–graphene oxide nanocomposites display percolation behavior influenced by water content, confirming that hydration and humidity can strongly affect charge transport and dielectric relaxation [35]. Broadband dielectric spectroscopy and scattering analyses of chitosan–graphene–silver metacomposites also support the relationship between nanoscale structure, conductive filler networks, and dielectric response [36]. Chitosan can also mediate nanoparticle assembly, as shown in studies of silver fractal formation in chitosan–Ag nanocomposites [37]. These examples show why processing route, moisture environment, filler distribution, and polymer–filler chemistry should be treated as core design parameters rather than secondary experimental details.

### 3.3. Debates and Unresolved Issues in Chitosan-Based Sustainable Electronics

A central debate is whether chitosan itself functions as an electronic material or whether its reported performance is mainly induced by added salts, oxides, conductive polymers, carbon materials, or metal nanoparticles. The evidence suggests that neither interpretation is sufficient on its own. Native chitosan contributes film-forming ability, ionic sites, functional groups, hydrogen-bonding structure, and interfacial compatibility, whereas added components provide charge carriers, localized states, percolation networks, or semiconducting domains. Green-synthesized carboxymethyl chitosan/boehmite and nanochitosan-reinforced PVA/cashew gum systems demonstrate that bio-based matrix design and filler reinforcement can jointly tune structural and functional properties [38,39]. Graphene oxide/silver nanocomposites and hydroxyapatite-reinforced PVA/chitosan blends further show that filler identity and dispersion can determine energy-storage and dielectric behavior [40,41].

A second unresolved issue concerns how conductivity, dielectric, and optical data should be interpreted. In chitosan-based nanocomposites, improved conductivity may arise from ion hopping, segmental chain motion, electrode polarization, interfacial polarization, percolation, localized states, filler-induced charge transfer, or a combination of these mechanisms. Hybrid Ag/Co_2_O_3_ nanofillers in PVP/chitosan blends and TiO_2_/Fe_2_O_3_-containing chitosan films show that mixed fillers can simultaneously alter optical absorption, dielectric response, and charge transport [42,43]. Cs/ZnO and Cs/ZnO/GO systems, together with starch/chitosan/NaTiO_3_ modified with ErCl_3_, reinforce the need to treat electronic properties as coupled structural and interfacial phenomena rather than as isolated material outputs [44,45].

A third debate concerns sustainability. Chitosan is renewable and biodegradable, but the sustainability of the final device depends on the complete material system. Some nanocomposites incorporate synthetic polymers, metal nanoparticles, rare-earth oxides, conductive polymers, or energy-intensive processing steps. A credible sustainable-electronics claim therefore requires attention to solvent choice, processing temperature, filler toxicity, device lifetime, recyclability, biodegradability, and end-of-life behavior. The theoretical challenge is to preserve the environmental advantages of chitosan while achieving electronic performance that is sufficient for practical green devices.

### 3.4. Structural Engineering and Processing Routes of Chitosan-Based Functional Films

The reviewed studies show that chitosan-based functional films are fabricated through diverse routes, including solution casting, spin coating, electrochemical deposition, electrospinning, electrospraying, sol–gel synthesis, and chemical doping. Solution-based processing is dominant because chitosan is compatible with aqueous or mildly acidic media and can be blended with dextran, starch, PVA, CMC, PVP, PCL, and other polymeric hosts. As summarized in Table 1, these processing routes are not merely preparative choices; they directly influence crystallinity, filler dispersion, electrolyte dissociation, and film morphology. Shukur et al. [9], Abdullah [25], and Abdulwahid et al. [26] showed that salt insertion and plasticization altered crystallinity and ion transport in chitosan-based electrolyte films. Abay [29] showed that spin-coated chitosan can function as an electronic interfacial layer, whereas Bonardd et al. [7] demonstrated that conductive polymer coating can transform chitosan into a flexible electronic substrate.

The structural diversity summarized in Table 1 also illustrates how chitosan supports multiple functional roles. Al-Mokaram et al. [46] used chitosan as a biosensor film or enzyme-immobilization layer, whereas AlAbduljabbar et al. [47] demonstrated its use in nanofiber-based functional membranes. Alharbi et al. [15] and Alhazime [12] showed that metal-oxide and mixed-oxide incorporation can reduce crystallite size, increase optical absorption, and improve dielectric behavior. Together, these studies indicate that chitosan-based films become electronically relevant when processing is used to control polymer–filler interactions, amorphous content, interfacial density, and conductive or ionic pathways.

Table 1 illustrates a representative structure → property → function cascade: incorporating ZnO and Al_2_O_3_ nanofillers into a chitosan/CMC blend reduces polymer-chain crystallinity and increases the amorphous fraction (**structure**), which raises the dielectric constant and narrows the optical band gap (**property**) [12], enabling use as a flexible dielectric capacitor and optoelectronic substrate (**function**). A second cascade appears in the rare-earth/oxide co-doped system: Ga_2_O_3_/Pr_6_O_11_ co-doping shrinks crystallite size from approximately 156 nm to 16 nm (**structure**), which improves thermal, optical, and dielectric performance simultaneously (**property**) [15], supporting use in solvent-processed dielectric films for electrical applications (**function**). These examples show that the processing routes summarized above act on structure first; structure, in turn, determines which properties and device roles become accessible.

### 3.5. Dielectric, Electrical, and Impedance Properties of Chitosan-Based Films and Polymer Electrolytes

The dielectric, electrical, and impedance literature identifies chitosan as a versatile host matrix for mobile ions, dipolar polarization, interfacial charge accumulation, and relaxation processes. In this group of studies, chitosan is commonly blended with methylcellulose, potato starch, PVA, dextran, rubber derivatives, or inorganic fillers and then modified using salts, plasticizers, crosslinkers, or ion-conducting additives. As summarized in Table 2, the electrical response of these systems is strongly governed by the balance between crystallinity reduction, amorphous-phase formation, salt dissociation, polymer-chain mobility, and electrode–electrolyte interfacial behavior. Abdullah et al. [30] showed that LiI-doped chitosan films exhibit conductivity behavior consistent with Jonscher’s power law, correlated barrier hopping, Arrhenius-type conduction, and non-Debye relaxation. Abdulwahid et al. [26,48] further demonstrated that ion transport in chitosan starch electrolytes depends on dopant concentration, ion speciation, structural disorder, and polymer-chain relaxation.

Plasticization appears to be one of the most effective strategies for improving ion transport in chitosan-based polymer electrolytes. Abdulwahid et al. [10] reported that a chitosan starch/NH_4_SCN electrolyte plasticized with 24 wt% glycerol reached a conductivity of 1.62 × 10^−3^ S/cm, an ionic transference number of 0.989, and electrochemical stability of 2.1 V. Similarly, Abdulwahid et al. [49] showed that glycerol-plasticized CS/KI biodegradable polymer electrolyte achieved an ionic transference number of 0.9375, stability of 2.50 V, and EDLC capacitance of 45.91 F/g. These findings indicate that plasticizers improve conductivity by increasing free volume, reducing crystallinity, enhancing segmental motion, and facilitating ion mobility. However, the improvement is not unlimited: excessive dopant or plasticizer loading may promote ion aggregation, block transport pathways, increase dielectric loss, or reduce mechanical integrity.

Across Table 2, the highest conductivity values were generally reported in systems where ion dissociation and polymer-chain flexibility were optimized together. Aziz et al. [50] reported a CS/LiClO_4_ polymer blend electrolyte with a conductivity of 5.16 × 10^−3^ S/cm, ionic transference number of 0.98, and electrochemical stability of approximately 2.3 V. Ahmed et al. [51] showed that glutaraldehyde-crosslinked PVA–CS alkaline anion-exchange membranes reached an ionic conductivity of 24.6 × 10^−3^ S/cm and a peak fuel-cell power density of 129.1 mW/cm^2^. Meanwhile, Alanazi and El Sayed [31] demonstrated that rare-earth-modified PVA–chitosan/NaTiO_3_ systems produced band-gap narrowing, enhanced dielectric response, and improved AC conductivity. Aziz et al. [52,53] also showed that glycerol-assisted Mg^2+^ and K^+^ transport can reduce interfacial resistance and support energy-storage performance. Overall, these studies confirm that impedance spectroscopy, dielectric analysis, electric modulus interpretation, and equivalent-circuit modeling are central tools for explaining structure-dependent charge transport in chitosan-based green electronic materials.

**Table 2:** Representative summary of studies on dielectric, electrical, and impedance properties of chitosan-based films and polymer electrolytes.

Reference	Material System	Dopant/Filler/Plasticizer	Electrical or Dielectric Method	Key Conductivity/ Dielectric Result	Proposed Transport Mechanism	Device Implication
[30]	CS biopolymer electrolyte	LiI salt	EIS, AC/DC conductivity, dielectric analysis, modulus analysis	Maximum ionic conductivity reached 6.26 × 10^−6^ S/cm at 40 wt% LiI	Jonscher power law, correlated barrier hopping, Arrhenius-type DC conduction, and non-Debye relaxation	Lithium-ion biopolymer electrolyte film for green electrochemical devices
[48]	CS starch proton-conducting electrolyte	NH_4_SCN salt	EIS, EEC modeling, dielectric analysis, modulus analysis	A 40 wt% dopant salt concentration produced the highest ion-conducting performance in the study	Proton relaxation coupled with polymer-chain dynamics	Flexible energy-storage electrolyte based on environmentally friendly biopolymers
[10]	CS starch/NH_4_SCN plasticized electrolyte	Glycerol	EIS, FTIR-derived ion transport analysis, TNM, LSV, CV, GCD	A 24 wt% glycerol sample produced conductivity of 1.62 × 10^−3^ S/cm, ionic transference number of 0.989, and electrochemical stability of 2.1 V	Plasticizer-induced crystallinity reduction and ion-mobility enhancement	EDLC electrolyte with capacitance of 16.1 F/g and 2500-cycle stability
[49]	CS/KI biodegradable polymer electrolyte	Glycerol	EIS, FTIR, TNM, LSV, CV	A 50 wt% glycerol electrolyte showed ionic transference number of 0.9375, electrochemical stability of 2.50 V, and EDLC capacitance of 45.91 F/g	Plasticizer-enhanced diffusion coefficient and carrier mobility	K^+^-conducting polymer membrane for EDLCs
[51]	PVA–CS alkaline anion-exchange membranes	Crosslinkers and glycerol	SEM, FTIR, ion-exchange capacity, ionic conductivity, fuel-cell testing	Glutaraldehyde-crosslinked membrane reached conductivity of 24.6 × 10^−3^ S/cm and ion-exchange capacity of 2.6 mmol/g	Crosslinking-mediated ionic pathway formation	Eco-friendly membrane for alkaline fuel cells with peak power density of 129.1 mW/cm^2^
[54]	Chitosan/chloroacetate chitosan blended with epoxidized natural rubber	ENR and crosslinking with NaOH/H_2_SO_4_	ATR-FTIR, AFM, LCR electrical testing	CS and CCS-based membranes showed the highest conductance, conductivity, capacitance, and dielectric constant; ENR-blended membranes (CCS10/ENR8, CS10/ENR8, CS15/ENR3) showed the highest resistance and resistivity	Functional-group and roughness-controlled proton-conducting behavior; CCS gave the lowest dissipation factor	Fuel-cell polymer electrolyte membrane with tunable electrical properties via ENR blending ratio
[31]	PVA–chitosan/NaTiO_3_ solid polymer electrolyte	Ga^3+^, Ce^3+^, Nd^3+^, or Er^3+^ salts	XRD, FESEM, EDS, FTIR, UV–Vis, TGA, dielectric spectroscopy	Band gap decreased from 4.4 to 3.5 eV; dielectric constant and AC conductivity improved	Salt dissociation, reduced crystallinity, and enhanced dielectric response	Flexible and thermally stable solid polymer electrolyte for supercapacitors
[50]	CS/LiClO_4_ polymer blend electrolyte	LiClO_4_	FTIR, XRD, FESEM, impedance analysis, TNM, LSV, CV	Highest conductivity reached 5.16 × 10^−3^ S/cm; ionic transference number was 0.98; electrochemical stability was approximately 2.3 V	Li^+^-dominated ion transport through the polymer blend electrolyte	High-conductivity electrolyte membrane for EDLCs
[53]	CS:PVA:Mg(NO_3_)_2_ polymer electrolyte	Glycerol	EIS, dielectric analysis, tanδ–Mʺ spectra, LSV	The 55 wt% glycerol sample showed the highest DC conductivity of 7.34 × 10^−5^ S/cm	Non-Debye relaxation and plasticizer-enhanced Mg^2+^ ion mobility	Magnesium-ion-conducting electrolyte for energy-storage applications
[52]	CS:PVA:KSCN biodegradable electrolyte	Glycerol	FTIR, impedance analysis, dielectric analysis, TNM, LSV, EDLC testing	Ionic transference number reached 0.952; electrochemical stability reached 2.85 V; charge-transfer resistance decreased with glycerol addition	Glycerol-assisted K^+^ transport and reduced interfacial resistance	Sustainable biodegradable electrolyte for high-energy-density EDLCs

Note: AC, alternating current; AFM, atomic force microscopy; ATR-FTIR, attenuated total reflectance Fourier-transform infrared spectroscopy; CCS, chloroacetate chitosan; CS, chitosan; CV, cyclic voltammetry; DC, direct current; EDLC, electric double-layer capacitor; EDS, energy-dispersive X-ray spectroscopy; EEC, electrical equivalent circuit; EIS, electrochemical impedance spectroscopy; ENR, epoxidized natural rubber; FESEM, field-emission scanning electron microscopy; FTIR, Fourier-transform infrared spectroscopy; GCD, galvanostatic charge–discharge; KSCN, potassium thiocyanate; LCR, inductance–capacitance–resistance; LSV, linear sweep voltammetry; PVA, poly(vinyl alcohol); SEM, scanning electron microscopy; TGA, thermogravimetric analysis; TNM, transference number measurement; UV–Vis, ultraviolet–visible spectroscopy; XRD, X-ray diffraction.

Table 2 traces two further structure → property → function cascades. Glutaraldehyde crosslinking of PVA–CS increases ion-exchange capacity and forms a more continuous ionic network (**structure**), which raises ionic conductivity to 24.6 × 10^−3^ S/cm (**property**) [51], enabling an alkaline-fuel-cell membrane with a peak power density of 129.1 mW/cm^2^ (**function**). Separately, increasing glycerol content in a chitosan–starch/NH_4_SCN electrolyte raises free volume and reduces crystallinity (**structure**), which lifts conductivity to 1.62 × 10^−3^ S/cm and ionic transference number to 0.989 (**property**) [10], supporting an EDLC electrolyte with stable capacitance over 2500 cycles (**function**). Both cascades confirm that the dielectric and electrical outcomes summarized in Table 2 are traceable consequences of a specific structural change, not properties intrinsic to chitosan itself.

### 3.6. Optoelectronic and Band-Gap Engineering in Chitosan-Based Nanocomposites

Optoelectronic studies show that chitosan can be transformed from a wide band-gap-insulating biopolymer into a tunable optical, dielectric, and semiconducting platform through controlled incorporation of metal oxides, quantum dots, metal–organic frameworks, rare-earth oxides, and conductive additives. As summarized in Table 3, the main function of these modifiers is not only to introduce new optical absorption centers but also to disrupt the semi-crystalline structure of chitosan, increase amorphous domains, create localized electronic states, and strengthen interfacial polarization. Abdalkarim et al. [17] demonstrated that FeMOF incorporation reduced the direct band gap of chitosan from 5.5 to 2.0 eV, while Abdalkarim et al. [16] showed that CrMOF integration reduced the band gap to approximately 3 eV and increased Urbach energy. These findings indicate that MOF-based chitosan composites can generate charge-transfer complexes and localized states that substantially modify optical transitions.

Metal-oxide-based systems provide another major route for optical and dielectric tuning. Ahmed [13] reported that ZrO_2_-reinforced chitosan nanocomposites showed band-gap narrowing from 5.4 to 2.9 eV, increased dielectric constant, and composition-dependent DC conductivity. Alanazi [14] similarly showed that α-Fe_2_O_3_-loaded PVA/chitosan films exhibited both direct and indirect band-gap reduction, together with a large increase in dielectric constant at low frequency. Alghamdi and Rajeh [55] demonstrated that ZnO nanorods in Cs/PVP films narrowed the optical band gap, increased refractive index and optical conductivity, and produced conductivity suitable for thin-film energy and optoelectronic applications. Alamdari et al. [19] further showed that flexible ZnO/CdWO_4_/chitosan mixed-oxide films generated blue-green and orange emissions, indicating the potential for flexible optical devices and intelligent textile-based sensing platforms.

The reviewed studies also show that optoelectronic improvement depends on an optimal balance between filler loading, interfacial coupling, transparency, dielectric response, and microstructural stability. Excessive filler addition may induce aggregation, optical scattering, dielectric loss, or reduced mechanical flexibility, whereas controlled dispersion can enhance absorption, refractive index, charge-carrier density, and dielectric polarization. Alsalmah et al. [32] showed that AgNO_3_ incorporation into PEG/chitosan films decreased the optical energy gap and increased dielectric and electrical parameters, while Domyati [42] reported substantial direct and indirect band-gap reductions in TiO_2_/Fe_2_O_3_-containing chitosan films. Together, these studies suggest that the optoelectronic performance of chitosan-based films is governed by a structure–property relationship in which filler chemistry, nanoscale dispersion, polymer–filler interactions, and localized electronic states determine whether the material functions as a dielectric substrate, optical coating, flexible optoelectronic film, or semiconducting nanocomposite.

**Table 3:** Representative summary of studies on optoelectronic and band-gap engineering in chitosan-based nanocomposites.

Reference	Chitosan-Based Optoelectronic System	Nanofiller or Modifier	Optical/Dielectric Characterization	Main Optical or Band-Gap Finding	Mechanistic Interpretation	Proposed Application
[17]	FeMOF-doped chitosan composite films	Fe(III)-MOF with captopril linker	FTIR, XRD, FESEM, DSC, UV–Vis, Tauc analysis	Direct band gap decreased from 5.5 to 2.0 eV; refractive index increased from 1.47 to 2.82; carrier density increased	Charge-transfer complexes and localized electronic states introduced by FeMOF	Biocompatible optoelectronics, solar cells, and biomedical devices
[16]	CrMOF-integrated chitosan films	Chromium(III)-MOF with captopril linker	FTIR, UV–Vis, TGA, DSC, BET, DLS, XRD, FESEM, EDX	Band gap decreased from 5.5 eV to approximately 3 eV; Urbach energy increased from 0.4394 to 0.9497 eV	MOF-induced localized states and optical dielectric modification	Optoelectronic polymer composites
[56]	Chitosan/PCL thin films hosting CdSe quantum dots	CdSe quantum dots	AC conductivity, dielectric constant, tanδ, Nyquist plots	CdSe incorporation altered electrical transport and dielectric relaxation	Hopping mechanism dominated charge-carrier transport	Semiconducting polymer-host network for optoelectronic and electrical applications
[13]	ZrO_2_-reinforced chitosan nanocomposites	ZrO_2_ nanoparticles	XRD, TGA, mechanical testing, optical and dielectric analysis	Band gap narrowed from 5.4 to 2.9 eV; dielectric constant reached ε′ ≈ 280 at 360 K; DC conductivity peaked at 5.1 × 10^−9^ S/cm (5.1 × 10^−7^ S/m)	Interfacial interactions, structural disruption, quantum confinement, and percolative pathways	UV-protective coatings, flexible electronics, and biomedical scaffolds
[14]	PVA/Cs–α-Fe_2_O_3_ nanocomposite films	α-Fe_2_O_3_ nanoparticles	XRD, FTIR, UV–Vis, dielectric spectroscopy	Direct band gap decreased from 5.76 to 4.68 eV; indirect band gap decreased from 5.03 to 3.34 eV; dielectric constant rose from 105 to 2656 at 10 Hz	Nanoceramic filler induced interfacial polarization and optical/electrical tunability	Flexible optoelectronic and organoelectronic devices
[55]	Cs/PVP–ZnO nanorod films	ZnO nanorods	XRD, TEM, FTIR, UV–Vis, photoluminescence, dielectric and modulus analysis	Band gap narrowed; optical conductivity and refractive index increased; PL peak appeared around 470 nm; conductivity reached 4.2 × 10^−5^ S/cm at 1.5 wt% ZnO	ZnO-induced amorphization, charge storage, and optical transition enhancement	Thin-film solar-cell and energy-storage applications
[19]	Flexible ZnO/CdWO_4_/chitosan mixed-oxide thin films	ZnO and CdWO_4_ mixed oxides	XRD, Raman, FESEM, TEM, photoluminescence, ionoluminescence	Films emitted in blue-green and orange regions; nanoparticles showed 20–80 nm size range	Interfacial charge transfer and tailored semiconductor band levels	Flexible optical devices and intelligent textile-based sensors
[57]	Nd_2_O_3_-doped chitosan films	Nd_2_O_3_ nanoparticles	SEM–EDX, XRD, molecular dynamics, optical analysis	Refractive index and dielectric constant increased while energy gap narrowed with Nd_2_O_3_ loading	Uniform nanoparticle adsorption and molecular interactions stabilized optically active structures	Optoelectronic and sensing materials
[32]	PEG/CS–AgNO_3_ nanocomposite films	AgNO_3_ nanoparticles	FTIR, XRD, optical, dielectric, and electrical measurements	Optical energy gap decreased; dielectric constant, dielectric loss, and conductivity increased with frequency	AgNO_3_ created localized states and increased amorphous character	Frequency-tunable nanodielectrics, flexible dielectric substrates, energy storage, and optoelectronics
[42]	Chitosan films containing TiO_2_/Fe_2_O_3_ nanoparticles	TiO_2_ and hematite nanoparticles prepared via laser ablation	XRD, FTIR, TGA, TEM, UV–Vis, AC measurements	Direct band gap decreased from 5.72 to 3.55 eV; indirect band gap decreased from 4.76 to 1.57 eV; refractive index increased from 2.04 to 2.95	Metal nanoparticle incorporation altered optical transitions and dielectric/optoelectronic response	New optoelectronic device materials

Note: AC, alternating current; BET, Brunauer–Emmett–Teller analysis; CS or Cs, chitosan; DSC, differential scanning calorimetry; DLS, dynamic light scattering; EDX, energy-dispersive X-ray spectroscopy; FESEM, field-emission scanning electron microscopy; FTIR, Fourier-transform infrared spectroscopy; MOF, metal–organic framework; PCL, poly(ε-caprolactone); PEG, polyethylene glycol; PL, photoluminescence; PVA, poly(vinyl alcohol); PVP, poly(vinylpyrrolidone); SEM, scanning electron microscopy; TGA, thermogravimetric analysis; UV–Vis, ultraviolet–visible spectroscopy; XRD, X-ray diffraction.

### 3.7. Electrochemical, Impedance-Sensing, and Smart-Device Applications

Electrochemical and impedance-sensing studies show that chitosan-based materials are particularly effective as interfacial platforms for analyte recognition, biomolecule immobilization, charge-transfer modulation, and signal amplification. Unlike polymer electrolyte studies, which primarily focus on ion transport through bulk films, sensor-oriented studies use chitosan to engineer the electrode–electrolyte interface. Chitosan provides amino and hydroxyl groups for immobilizing enzymes, antibodies, nanoparticles, conductive polymers, carbon materials, and metal oxides, while its film-forming ability helps stabilize active layers on electrodes or flexible substrates. As summarized in Table 4, these systems have been applied to impedimetric immunosensors, electrochemical drug sensors, pesticide detection, gas sensors, formaldehyde sensors, sulfite detection, and smart-label platforms.

Impedance-based biosensors provide some of the clearest evidence of chitosan’s role in interfacial charge-transfer control. Afkhami et al. [58] developed an impedimetric immunosensor using a Au nanoparticles/graphene–chitosan composite for label-free detection of botulinum neurotoxin A. In that system, chitosan supported antibody immobilization and helped stabilize the nanocomposite interface, while impedance changes reflected restricted electron transfer after antigen–antibody interaction. Aggas et al. [18] further demonstrated that chitosan can operate as a matrix for polyaniline-based bio-nanocomposites, producing chemiresistive and chemicapacitive devices whose electrical behavior depends on morphology, composition, and percolation threshold. These studies show that chitosan does not simply hold active materials in place; it contributes to interfacial organization, hydration-dependent transport, and impedance modulation.

Electrochemical sensors also demonstrate the functional adaptability of chitosan-based interfaces. Abd-Elsabour et al. [59] reported a chitosan curcumin Schiff base/NiO nanorod-modified electrode for nanomolar detection of 5-fluorouracil, while Aghris et al. [60] used a chitosan-coated graphite electrode for selective flubendiamide detection in food and water samples. Saisa et al. [61] developed a chitosan–ZnO nanoparticle-based sensor for formaldehyde detection. Akanbi et al. [62] further extended chitosan-containing nanocomposite electrodes toward field-oriented plant-disease sensing by combining AuNPs, chitosan nanoparticles, and MWCNTs on a screen-printed electrode for quinoline detection in *G. boninense*-infected plants. Ahmed et al. [63] combined chitosan-coated gold nanoparticles with machine-learning-assisted electrochemical modeling for ciprofloxacin quantification. These examples indicate that chitosan-based films can support sensitive analytical performance when combined with conductive nanoparticles, catalytic oxides, carbon materials, or computational optimization.

Beyond conventional electrochemical and impedimetric platforms, chitosan-based conductive composites have also been extended toward smart-device interfaces. Abdollahi-Esfahlani et al. [64] fabricated PVA/chitosan/polyaniline nanofibrous membranes as dual chemiresistive ammonia sensors and smart green labels for fish freshness monitoring, illustrating how chitosan-containing conductive architectures can support broader responsive sensing applications. Adeosun et al. [65] showed that conductive polypyrrole–chitosan thin films can detect sulfite in food and biological samples, whereas Akhter et al. [66] demonstrated that functionalized MWCNT–chitosan films can enhance amperometric paracetamol detection. Ali et al. [67] extended this gas-sensing role to a WO_3_-nanoparticle/chitosan-glycerol hybrid membrane, in which the ionic-liquid-plasticized chitosan matrix had control over membrane conductivity and enabled a fast, selective, low-temperature H_2_S sensor. Across these studies, the main design principle is the construction of a stable, conductive, and chemically interactive chitosan-based interface. However, device translation remains limited by incomplete reporting of humidity effects, film thickness, long-term stability, reproducibility, real-sample validation, and mechanical durability under flexible-use conditions.

**Table 4:** Representative summary of studies on electrochemical, impedance-sensing, and smart-device applications of chitosan-based functional films and nanocomposites.

Reference	Chitosan-Based Sensing System	Target Analyte or Function	Electrochemical/Sensing Method	Key Performance Result	Functional Role of Chitosan	Device Relevance
[58]	Au nanoparticles/graphene–chitosan composite immunosensor	Botulinum neurotoxin serotype A	EIS, CV, SEM, TEM, AFM, XRD, FTIR	Linear response from ~1.8 fM to ~1.8 pM (0.27 to 268 pg/mL) with LOD of ~0.7 fM (0.11 pg/mL; converted using a ~150 kDa toxin molar mass)	Stabilized AuNP/graphene interface and supported antibody immobilization	Label-free impedimetric biosensor for toxin detection
[18]	Polyaniline-chloride nanofibers in chitosan matrix	Chemiresistive and chemicapacitive behavior	Four-probe conductivity, CV, EIS, modified Randles modeling	Conductivity depended on composition; percolation threshold was approximately 30 wt%; 50:50 composition was favored	Provided bio-nanocomposite matrix and morphology control	Biosensor and biocircuit elements with tunable resistive/capacitive response
[59]	Chitosan curcumin Schiff base-decorated NiO nanorods on GCE	5-Fluorouracil	CV, DPV, chronoamperometry	Linear range of 0.1–150.0 μM with LOD of 21.75 nM	Organic functional layer for hybrid electrochemical interface	Sensitive drug sensor for serum and urine samples
[60]	Chitosan-coated pencil graphite electrode	Flubendiamide insecticide	CV and DPV	Linear range from 5.0 × 10^−7^ to 1.0 × 10^−5^ mol/L with LOD of 5.0 × 10^−8^ mol/L	Biopolymer coating improved electrode selectivity, stability, and reproducibility	Food and surface-water pesticide detection
[61]	Chitosan–ZnO nanoparticle sensor on copper screen-printed electrode	Formaldehyde	CV	LOD of 0.18 nM, LOQ of 0.59 nM, and linear range of 1–100 × 10^−5^ μM (corrected from the non-standard “nM/L”/“μM/L” units used in the original submission)	Film-forming matrix for ZnO nanoparticle-based sensing layer	Low-cost formaldehyde sensor
[63]	AuNPs/chitosan-modified glassy carbon electrode with ML-assisted optimization	Ciprofloxacin	DPV, electrochemical modeling, machine-learning prediction	CatBoost model achieved R^2^ = 0.9985; LOD was 0.049 μM; sensitivity was 786.72 μA μM^−1^ cm^−2^	Stabilized AuNP interface and supported electrochemical signal generation	Ultrasensitive antibiotic monitoring in water samples
[65]	Conductive polypyrrole–chitosan thin film	Sulfite	Electrochemical synthesis, DPV	LOD of 0.21 μM, sensitivity of 15.28 μA μM^−1^ cm^−2^, and linearity from 50 to 1100 μM	Conductive biopolymer film forming an electrocatalytic interface	Sulfite detection in food and biological samples
[66]	Functionalized MWCNT–chitosan–co-modified GCE	Paracetamol	CV, EIS, DPV	Linear range from 0.1 to 400 μmol/L with LOD of 0.01 μmol/L	Matrix for immobilizing Co on f-MWCNTs and enhancing electrocatalytic activity	Pharmaceutical and serum-sample analysis
[62]	AuNPs/chitosan nanoparticles/MWCNT-modified screen-printed electrode	Quinoline	CV and LSV	Linear range from 0.0004 to 1.0 μM with LOD of 3.75 nM	Supported layer-by-layer nanocomposite assembly and improved electrode stability	Field detection of quinoline in infected plants
[64]	PVA/chitosan/polyaniline nanofibrous membrane	Ammonia gas and fish freshness monitoring	Chemiresistive sensing and colorimetric smart-label response	Linear response from 25 to 100 ppm NH_3_; response/recovery time of 150/18 s at 100 ppm; LOD of 571 ppb	Biocompatible nanofibrous host for PANI deposition and responsive gas interaction	Dual chemiresistive sensor and smart green freshness label
[67]	WO_3_-nanoparticle/chitosan-glycerol hybrid membrane	Hydrogen sulfide (H_2_S) gas	Chemiresistive gas sensing at controlled temperature	Fast response of 13.6 ± 1.7 s and sensitivity of 15 ppm at a low operating temperature of 40 °C, with low humidity dependence	Conductivity-controlling, ionic-liquid-plasticized membrane host for WO_3_ nanoparticles	Flexible, low-power organic H_2_S sensor for next-generation organic electronics

Note: AFM, atomic force microscopy; AuNPs, gold nanoparticles; CV, cyclic voltammetry; DPV, differential pulse voltammetry; EIS, electrochemical impedance spectroscopy; FTIR, Fourier-transform infrared spectroscopy; GCE, glassy carbon electrode; LOD, limit of detection; LOQ, limit of quantification; LSV, linear sweep voltammetry; ML, machine learning; MWCNT, multi-walled carbon nanotube; PANI, polyaniline; PVA, poly(vinyl alcohol); SEM, scanning electron microscopy; TEM, transmission electron microscopy; XRD, X-ray diffraction.

Table 1, Table 2, Table 3 and Table 4 report detailed qualitative and quantitative findings for each individual study, but the four modification strategies they cover are not directly compared against one another on a common set of performance metrics. Table 5 addresses this by distilling the best-in-class values reported across Table 1, Table 2, Table 3 and Table 4 and Section 3.9 for the four performance metrics most often used to compare chitosan-based systems, cross-tabulated against the four dominant modification strategies identified in this review.

Table 5 makes the trade-offs among strategies explicit: salt-doped and crosslinked electrolytes deliver the highest bulk ionic conductivities, MOF- and metal-oxide-based systems deliver the largest and most tunable band-gap and dielectric responses, and carbon-based interfacial architectures deliver the lowest detection limits. The optimal modification route therefore depends on which functional domain, energy storage, optoelectronics, or sensing that a given application targets, a point developed further in the prescriptive structure–property control table (Table 6) below.

### 3.8. Integrated Structure–Property–Function Framework

The evidence synthesized in this review supports the interpretation that chitosan-based films and nanocomposites should be understood as structure-sensitive functional systems rather than as generic biodegradable matrices. Their electronic and device-level performance is shaped by the combined effects of chitosan molecular chemistry, processing route, filler dispersion, crystallinity, amorphous fraction, interfacial polarization, and device architecture. This interpretation explains why the same material family can appear in different functional contexts, including polymer electrolytes, optoelectronic films, dielectric substrates, electrochemical sensors, impedance devices, and gas or humidity sensors. In electrolyte studies, performance is commonly evaluated through ionic conductivity, ion transference number, electrochemical stability, dielectric relaxation, and impedance fitting. Optoelectronic studies, by contrast, emphasize band-gap narrowing, refractive index, optical conductivity, Urbach energy, photoluminescence, and dielectric response. Sensor-oriented studies usually prioritize the limit of detection, linear range, response and recovery time, selectivity, stability, and real-sample validation. These outcomes should not be treated as unrelated observations. They reflect different ways in which charge transport, interfacial engineering, and structure-dependent functional behavior emerge in chitosan-based systems.

These relationships are summarized in Figure 2, which presents the structure–property–function framework developed from the reviewed literature. Material inputs, including chitosan, chitosan derivatives, nanocomposite fillers, salts, plasticizers, conductive polymers, and other functional additives, establish the chemical and structural possibilities of each system. Processing and modification strategies then determine how these components are assembled into films, membranes, coatings, or interfacial layers. Structural characteristics such as crystallinity, morphology, porosity, filler distribution, crosslinking density, hydration state, and interfacial organization subsequently govern the measurable electrical, optical, dielectric, electrochemical, or sensing properties of the final material. Figure 2 therefore serves as an interpretive map for understanding why similar chitosan-based compositions may produce different functional outcomes when processing conditions or device configurations differ.

The framework also clarifies why application performance cannot be attributed to material composition alone. A chitosan film containing a given filler may show different behavior depending on filler dispersion, solvent environment, film thickness, drying conditions, crosslinking density, processing temperature, electrode contact, and residual moisture. Similarly, improved conductivity, dielectric response, optical absorption, or sensing performance may arise from several coupled mechanisms rather than from one isolated factor. These mechanisms include ion hopping, segmental chain motion, interfacial polarization, localized electronic states, percolation pathways, electrode polarization, and analyte-induced swelling or charge transfer. This point is important for interpreting the literature because studies using broadly similar chitosan-based formulations often report different performance levels. The variation is not necessarily contradictory; it may reflect differences in structure, interface quality, environmental control, or measurement protocol.

The reviewed evidence shows that chitosan’s native chemistry provides a useful functional starting point, but high device performance usually requires targeted modification. Salt-doped and plasticized systems demonstrate that ion transport depends on the balance among amorphization, salt dissociation, ion aggregation, free volume, and polymer-chain mobility. MOF- and metal-oxide-based systems show that optical and dielectric responses can be tuned through localized electronic states, interfacial polarization, semiconducting filler domains, and changes in crystallinity. Conductive polymer and carbon-based systems further indicate that percolation pathways and microstructural continuity are critical for resistive and capacitive behavior. In sensing applications, chitosan contributes not only as a support matrix but also as an immobilization medium, swelling-responsive layer, interfacial binder, and transduction-supporting platform. Impedimetric and electrochemical studies have reported low detection limits for botulinum neurotoxin A, flubendiamide, formaldehyde, ciprofloxacin, 5-fluorouracil, sulfite, quinoline, ammonia, paracetamol, and glucose using chitosan-containing sensing architectures [58,59,60,61,63,65,69]. These examples support the view that chitosan is most effective when its chemical functionality is integrated with conductive, catalytic, optical, or recognition components.

To give Figure 2 predictive rather than purely descriptive value, Table 6 converts the strategy-by-strategy structural levers identified above into an explicit structure–property control table: for each structural parameter, it states which property that parameter most strongly governs, the direction of the effect observed in the reviewed corpus, a recommended composition or processing window, and the principal trade-off that must be managed when that parameter is pushed too far. Read together with Table 5, Table 6 is intended to let a researcher move from “which strategy gives the best value of metric X” to “which structural parameter should I adjust, and by how much, to move a given system toward that value”.

**Table 6:** Prescriptive structure–property control table linking structural parameters to the properties they govern, evidence from the reviewed corpus, recommended composition/processing windows, and the associated performance trade-offs.

Structural Parameter	Property Most Strongly Controlled	Direction of Effect (Evidence)	Recommended Composition/ Processing Window	Trade-Off to Manage
Crystallinity/amorphous fraction	Ionic conductivity; optical band gap	Increasing amorphous fraction, via plasticization, salt doping, or nanofiller-induced disruption, raises ion mobility and narrows the optical band gap [10,12,13,15,25,26]	Moderate plasticizer or salt loading sufficient to disrupt crystallinity without excessive swelling, e.g., the 24–50 wt% glycerol range that produced conductivity and EDLC-capacitance gains in the CS-starch/NH_4_SCN and CS/KI systems reviewed here [10,49]	Excessive amorphization promotes ion aggregation and reduces mechanical integrity
Filler loading and dispersion (nano-oxide, MOF, carbon)	Dielectric constant; band-gap narrowing; percolation-dependent conductivity	Below the percolation threshold (~30 wt% PANI in the reviewed polyaniline–chitosan system) behavior is capacitive; above it, behavior becomes increasingly resistive [18]	Operate near but below the percolation threshold for capacitive/sensing behavior, or above it for chemiresistive behavior, matched to the intended device role	Overloading causes filler aggregation, optical scattering, and dielectric loss
Crosslink density (covalent or ionic network formation)	Ion-exchange capacity; humidity/water-uptake stability	Glutaraldehyde crosslinking raised ion-exchange capacity and conductivity to 24.6 × 10^−3^ S/cm, the highest value in this corpus [51]; crosslinking-based environmental/water-stability benefits are discussed further in Section 3.9 [70,71]	Moderate covalent crosslinking that forms continuous ionic pathways without excessively restricting chain mobility	Over-crosslinking reduces segmental mobility and can lower ionic conductivity
Interfacial/electrode architecture (nanoparticle-conjugate immobilization layers)	Limit of detection; sensitivity in sensing devices	Layer-by-layer nanocomposite assembly (AuNPs/MWCNT) achieved an LOD of 3.75 nM [62]; a graphene/AuNP interface achieved sub-picomolar LOD [58]	Multi-component conductive interfacial layering, combining a metal nanoparticle, a carbon material, and a chitosan binder, to maximize signal transduction	Added architectural complexity can reduce reproducibility and fabrication scalability
Salt/dopant chemistry and concentration	Ionic conductivity and, in doped optoelectronic films, band gap	Loading alone does not predict performance: 40 wt% LiI still reached only 6.26 × 10^−6^ S/cm [30], while lower-loading LiClO_4_ and glutaraldehyde-crosslinked systems reached above 10^−3^ S/cm [50,51], indicating that dissociation efficiency and matrix compatibility matter more than nominal salt content	Prioritize salts/dopants with high dissociation constants and matrix compatibility over simply maximizing weight fraction	High salt loading beyond the optimum promotes ion-pair aggregation, which can reduce, not increase, net conductivity

Note: AuNPs, gold nanoparticles; EDLC, electric double-layer capacitor; LOD, limit of detection; MOF, metal–organic framework; MWCNT, multi-walled carbon nanotube; PANI, polyaniline. Entries are drawn exclusively from the primary studies already summarized in Table 1, Table 2, Table 3 and Table 4 and Section 3.9 of this review; no additional literature search was conducted to populate this table.

Despite these advances, cross-study comparison remains difficult because reporting practices are uneven. Many studies use extensive characterization, including FTIR, XRD, SEM or FESEM, AFM, TEM, EDX, TGA, DSC, UV–Vis spectroscopy, dielectric spectroscopy, CV, DPV, EIS, electric modulus analysis, and equivalent-circuit fitting. These methods are appropriate for establishing structure–property relationships, but their value is reduced when essential experimental parameters are missing. Chitosan source, molecular weight, degree of deacetylation, concentration, solvent condition, film thickness, drying protocol, humidity, number of replicates, and long-term stability are not always reported consistently. These omissions are scientifically important because chitosan is sensitive to protonation state, hydration, molecular weight distribution, and processing history. Humidity is particularly critical: water can enhance proton or ion transport, alter dielectric relaxation, change swelling behavior, and destabilize the response of sensors or polymer electrolytes. Without careful reporting of these variables, conductivity, dielectric, optical, and sensing data remain difficult to compare across studies.

Part of the apparent scatter in Table 2’s conductivity values reflects genuinely different, competing ionic charge-transport mechanisms rather than a single “conductivity” quantity that simply scales with salt or plasticizer content. Abdullah et al. [30] explicitly interpret their LiI-doped chitosan films in terms of Jonscher’s universal power law and correlated barrier hopping among localized (coordination) states, together with an Arrhenius-type thermally activated direct-current component and non-Debye relaxation; Aziz et al. [53] similarly use electric-modulus and tan δ–M″ spectral analysis to resolve non-Debye relaxation behavior in a Mg^2+^-conducting system, while Aziz et al. [52] report comparable glycerol-assisted K^+^-transport behavior, with an ionic transference number of 0.952 confirming that conduction is overwhelmingly ionic rather than electronic. All three systems are therefore ionic conductors in which the mobile cation (Li^+^, Mg^2+^, or K^+^) hops among localized coordination sites in the polymer matrix, a correlated-hopping regime rather than an electronic or small-polaron one. By contrast, the highest-conductivity systems in Table 2, the glutaraldehyde-crosslinked PVA–CS membrane [51] and the glycerol-plasticized CS-starch/NH_4_SCN electrolyte [10], are reported in terms of ionic transference number and ion-exchange capacity, consistent with a vehicular, ion-dissociation-dominated ionic transport regime rather than correlated hopping among fixed sites. Because hopping-type and ion-dissociation-dominated transport have fundamentally different frequency and temperature dependence, two chitosan-salt systems that look nominally similar in composition can report conductivities that differ by three orders of magnitude, from 6.24–6.26 × 10^−6^ S/cm [9,30] to 24.6 × 10^−3^ S/cm [51], partly because they are not operating through the same microscopic mechanism, not only because one formulation is intrinsically “better” than the other.

A related, and largely unaddressed, source of apparent variability is electrode polarization at the film–electrode interface. Very large low-frequency dielectric constants reported for filler-modified chitosan systems, such as ε′ ≈ 2656 at 10 Hz for the PVA/Cs–α-Fe_2_O_3_ system [14] and ε′ ≈ 280 at 360 K for the ZrO_2_–chitosan system [13], are difficult to interpret as purely intrinsic bulk permittivity. At low measurement frequencies, mobile-ion accumulation and Maxwell–Wagner–Sillars-type interfacial polarization at a blocking metal electrode can dominate the apparent capacitance, inflating the extracted ε′ well above the material’s true bulk dielectric response. Electric-modulus analysis (M* = 1/ε*), already used by several of the reviewed studies for relaxation-time extraction [30,48,53], is the standard tool for deconvolving genuine bulk relaxation from electrode-interface polarization, but none of the reviewed dielectric studies report having applied it specifically for this purpose. The peak dielectric-constant values summarized in Table 5 should therefore be read as apparent, electrode-polarization-inclusive values rather than as directly comparable intrinsic bulk permittivities, and this ambiguity is itself a concrete, addressable target for future reporting standards in this field.

Uncontrolled ambient humidity during electrical and dielectric testing compounds both effects. Because chitosan’s hydrogen-bonded matrix absorbs ambient moisture readily (Section 3.9), adsorbed water can introduce an additional, parallel proton- or ion-conduction pathway during measurement. None of the studies summarized in Table 2 report the relative humidity or environmental control condition under which conductivity or dielectric measurements were performed. Consequently, part of the order-of-magnitude spread in Table 2 and Table 5 may reflect differences in ambient testing humidity at the time of measurement rather than genuine differences in material design, reinforcing the case made in Section 3.9 for standardized, humidity-controlled reporting across future chitosan-based electronics studies.

As an integrative example, Table 4’s polyaniline–chitosan system shows the same structural lever operating across functional domains: increasing polyaniline loading toward the approximately 30 wt% percolation threshold (**structure**) shifts the film from predominantly capacitive to increasingly resistive electrical behavior (**property**) [18], a shift that is deliberately exploited to configure the resulting device as either a chemiresistive biosensor or a chemicapacitive bioelectronic circuit element (**function**). This cross-thematic cascade demonstrates that the structure–property–function logic traced separately for Table 1 and Table 2 above also governs sensor-oriented systems, reinforcing Figure 2’s central claim that composition, processing, and structure jointly determine device role, rather than device role being an intrinsic property of chitosan itself.

### 3.9. Research Trends, Challenges, and Future Directions

The main findings, research trends, challenges, and future directions emerging from this synthesis are organized in Figure 3. The figure brings together the major evidence patterns identified in this review: the growing use of chitosan-based systems in flexible electronics, bio-derived sensors, polymer electrolytes, optoelectronic films, packaging-related devices, and hybrid nanocomposites; and the persistent limitations related to material standardization, scalability, device validation, and sustainability assessment. Figure 3 is therefore not only a summary of trends. It also identifies where the field remains methodologically weak. In many cases, studies report enhanced conductivity, dielectric constant, optical response, or sensing performance without fully documenting the material and environmental parameters that control those improvements. As a result, it is often difficult to determine whether improved performance arises mainly from chitosan chemistry, filler effects, processing-induced morphology, interfacial structure, or measurement conditions.

Several design trade-offs are apparent across the reviewed studies. Increasing filler loading can reduce the optical band gap and enhance dielectric response, but excessive filler content may lead to aggregation, optical opacity, dielectric loss, brittleness, or blocked ion pathways. Plasticizers can improve ionic mobility, but they may reduce mechanical stability, dimensional integrity, or long-term durability. Conductive polymers and carbon-based fillers can increase charge transport, although their inclusion may complicate biodegradability claims and end-of-life management. Noble metals, metal oxides, rare-earth compounds, and MOFs often produce strong functional improvements, but they also raise concerns related to cost, toxicity, resource availability, recyclability, and processing energy. These trade-offs show that a material should not be described as sustainable only because it contains chitosan. Sustainability needs to be assessed at the level of the complete material system, including filler selection, solvent use, fabrication conditions, device lifetime, recyclability, and environmental fate.

Humidity is a distinct and pervasive stability challenge for chitosan-based devices, because the same amino/hydroxyl-rich, hydrogen-bonded structure that gives chitosan its ion-transport and biodegradability advantages also makes it intrinsically hygroscopic. Ambient moisture uptake plasticizes the polymer matrix, increases free-volume and segmental mobility, and adds ionic and interfacial polarization pathways, so dielectric constant, loss, and apparent conductivity can drift substantially with relative humidity even when the underlying composition is unchanged, compounding the cross-study comparability problem discussed in Section 3.8. Within the reviewed corpus, several crosslinked or surface-modified chitosan architectures are relevant to this question, though with uneven directness of evidence. An ionically crosslinked chitosan hydrogel membrane electrolyte was developed for long-lived electrical double-layer capacitors [70], and a chemically crosslinked chitosan dielectric thin film provides the strongest direct evidence: crosslinked films are markedly more hydrophobic than native chitosan and show enhanced environmental and water stability while retaining strong electrical performance [71]. A conducting-polymer-modified chitin-nanocrystal layer developed for triboelectric energy harvesting [72] and a physically crosslinked, gel-gated transistor developed for hydrogen-sulfide gas sensing [73] use related crosslinking or surface-modification approaches but were not framed by their authors as dedicated humidity- or vapor-stability studies, so the evidence for crosslinking as a general humidity-mitigation strategy beyond [71] should be read as suggestive rather than established. Complementary approaches used to control moisture ingress in other biopolymer and hydrogel electronic systems—hydrophobic surface coatings, fluorination of surface functional groups, and thin hybrid encapsulation layers—are largely absent from the chitosan-based electronics studies identified in this review and represent an actionable direction for future work: pairing chitosan’s intrinsic ion-transport and biodegradability advantages with a thin, removable, or biodegradable moisture barrier (e.g., a hydrophobic wax, silane, or fluoropolymer overlayer) could decouple bulk conductive and dielectric performance from ambient humidity fluctuations, extending operational device lifetime without compromising end-of-life degradability.

Beyond the well-established chitosan/metal-oxide and chitosan/MOF systems discussed above, our screening specifically searched the literature corpus for three under-represented directions: intrinsically conductive chitosan, stretchable/wearable chitosan electronics, and self-healing chitosan-based electronic skin. Rather than list these as three unconnected emerging topics, each is framed below as a logical extension of the structure–property–function logic developed in Section 3.8 (Figure 2, Table 6): a target function is stated, the structural adjustment that the reviewed evidence suggests is needed to reach it, and the specific knowledge gap currently holding that extension back is identified.

For intrinsically conductive chitosan, the target function is bulk electronic conductivity high enough for chitosan to function as an active conductive element rather than merely as a host or coating substrate. Eleven studies in this corpus report progress toward this target, including a graphite-reinforced natural-rubber/chitosan composite reaching electrical conductivity roughly six orders of magnitude above the unfilled polymer [68], a chitosan/carbon-nanotube–graphene-oxide film with charge-carrier mobility up to approximately 11 cm^2^ V^−1^ s^−1^ [74], and a chitosan-succinamide field-effect-transistor sensor platform [75]. The structural adjustment implied by Table 6’s filler-loading/dispersion lever is to push conductive-filler incorporation from the surface-coating architectures used for interfacial modification in Table 1 (P3HT- and PPy-coated chitosan films [7,46]) toward continuous, percolating conductive networks integrated throughout the bulk chitosan matrix, analogous to the percolation behavior already characterized for the polyaniline–chitosan sensing system [18]. The corresponding knowledge gap is that none of the eleven studies identified here report the crosslink-density/humidity-stability trade-off established in Table 6 for this same structural lever, so it remains unknown whether percolating conductive chitosan networks can retain stable conductivity under the ambient-humidity conditions flagged as a stability challenge earlier in this section.

For stretchable and wearable chitosan electronics, the target function is retaining ionic or electronic conductivity under large, repeated mechanical deformation rather than under the static film geometries evaluated in Table 1, Table 2, Table 3 and Table 4. Twenty-four studies address this direction, including a chitin-nanofiber composite hydrogel reported as simultaneously highly stretchable, tough, and electrically conductive for wearable-sensor use [76], a graphite/chitosan-ink strain sensor with a gauge factor of 5.44 over an 83% strain range [77], and an ionic-liquid-exfoliated graphene/chitosan hydrogel stretchable to 2250% strain with a gauge factor of 12.31 [78]. The structural adjustment implied here is an extension of Table 6’s crystallinity/amorphous-fraction lever: the same amorphization and plasticization mechanisms shown to raise ion mobility in Table 2’s electrolyte systems [10,25,49] also need to be tuned for elastomeric, reversibly deformable network structure rather than for dimensional stability in a rigid cast film. The corresponding knowledge gap is that none of the twenty-four studies identified here report long-term cyclic mechanical-fatigue data alongside electrical performance, so whether these gauge factors and conductivities are retained over the thousands of strain cycles that a wearable device would experience in practice remains unestablished.

For self-healing chitosan-based electronic skin, the target function is autonomous recovery of both structural integrity and electrical continuity after mechanical damage, which would extend the crosslink-density lever in Table 6 toward reversible, dynamic bonding chemistries (e.g., dynamic imine, hydrogen-bonded, or ionic-crosslink networks) rather than the fixed covalent crosslinking used to stabilize conductivity and humidity resistance in the systems reviewed in Table 2 and Table 6 [51,70,71]. Unlike the two directions above, our search corpus did not return any chitosan-based study reporting self-healing behavior in an electronic-skin or comparable device context. Self-healing chitosan-based e-skin is therefore reported here as an identified structural target with no supporting primary study in the evidence base assembled for this review, rather than as a synthesized finding, and the knowledge gap is correspondingly the most fundamental of the three: whether reversible dynamic-bond chemistries compatible with chitosan’s amino/hydroxyl functionality can restore electrical continuity, not only mechanical integrity, after damage has not yet been demonstrated in the reviewed literature.

Figure 4 translates these findings into an integrated design-to-application pathway for chitosan-based nanocomposite materials, coupled with the sustainability considerations that determine whether a laboratory-scale result translates into a viable device. The pathway begins with material formulation, including the selection of chitosan, chitosan derivatives, fillers, dopants, ionic species, plasticizers, crosslinkers, and conductive or catalytic components. These materials are then processed through routes such as solution casting, electrospinning, coating, blending, electrodeposition, sol–gel processing, or green chemical modification. The resulting structure determines the electrical, optical, dielectric, electrochemical, mechanical, barrier, and sensing properties that define suitability for specific applications. This pathway is useful because many studies in the field still focus on improving a single property, such as conductivity, optical absorption, dielectric constant, or sensing response, whereas practical devices require several properties to be optimized at the same time: a polymer electrolyte must combine conductivity with electrochemical stability and mechanical integrity, an optoelectronic film must balance band-gap tuning with transparency, flexibility, and dielectric loss, and a sensor must combine sensitivity with selectivity, recovery, stability, and real-sample performance. Critically, each stage of this pathway feeds back into a sustainability and translation loop spanning manufacturability (scalability, reproducibility, process energy), reliability (long-term stability, humidity and cycling durability), environmental burden (solvent choice, filler toxicity, life-cycle impact), and end-of-life behavior (recyclability, biodegradability of the full material system, not chitosan alone). A material cannot be judged sustainable solely by its conductivity, dielectric constant, band gap, or detection limit; it must also be evaluated against this full loop before the next generation of material-formulation choices is made.

The practical implication is that future studies should move beyond proof-of-concept demonstrations toward standardized and reproducible design rules. For polymer electrolytes, research should harmonize impedance fitting, ion transference measurements, electrochemical stability windows, humidity control, and cycling conditions. For optoelectronic films, studies should report not only band-gap modification and dielectric response but also transparency, flexibility, mechanical integrity, thermal stability, and operational durability. For sensors, real-sample validation, long-term stability, recovery behavior, cross-sensitivity, calibration robustness, and environmental tolerance should become routine. Machine-learning-assisted optimization, as demonstrated in recent electrochemical sensing work, may help relate processing variables and material descriptors to device performance more systematically [63]. Molecular-level engineering of molecular weight, degree of deacetylation, and protonation state, together with computational and machine-learning-assisted design linking formulation and processing variables to performance, are needed to move beyond trial-and-error experimentation; both are useful only when the underlying experimental data are well reported and comparable.

The roadmap also points to the need for stronger validation under realistic operating conditions. Many chitosan-based systems are evaluated under controlled laboratory settings, but fewer studies examine long-term stability, humidity sensitivity, repeated cycling, mechanical deformation, fouling, storage effects, or performance in complex sample matrices. This gap is especially important for sensors, polymer electrolytes, and flexible devices, where environmental exposure can strongly alter material response. Benchmarking against established synthetic materials is also needed to clarify where chitosan-based systems offer genuine advantages and where their performance remains insufficient.

Taken together, Figure 2, Figure 3 and Figure 4 describe an integrated design logic for chitosan-based sustainable electronics: Figure 2 establishes the relationship among structure, properties, and function; Figure 3 summarizes the main findings, trends, emerging and under-represented directions, and challenges; and Figure 4 translates the evidence into a design-to-application pathway coupled with the sustainability feedback loop needed for field maturation. Across these figures, the main message is consistent: chitosan-based films and nanocomposites have strong potential as sustainable functional materials, but their practical value depends on coordinated optimization of composition, processing, structure, performance, stability, and environmental responsibility. The contribution of this review is therefore not limited to cataloguing applications of chitosan-based materials; it provides an integrated interpretation of how these materials become functional electronic systems, in which electronic, dielectric, optoelectronic, electrochemical, and sensing performance emerges from the interplay among polymer chemistry, filler architecture, interfacial organization, environmental conditions, and device context. Future progress will depend on studies that report material parameters transparently, evaluate device performance under realistic conditions, and assess sustainability at the full-system level, allowing chitosan-based films and nanocomposites to move from laboratory-scale demonstrations toward more scalable, reliable, and environmentally accountable technologies.

## 4. Conclusions

This systematic review shows that chitosan-based films and nanocomposites have developed into a broad class of functional materials for sustainable electronics. Across 156 included studies, chitosan was used not only as a biodegradable film-forming matrix but also as a chemically active platform for ion coordination, filler stabilization, interfacial adhesion, charge transport, polarization, optical tuning, and analyte interaction. The reviewed evidence confirms that the functional performance of chitosan-based systems depends strongly on the relationship among composition, processing, structure, and device context.

Four main findings emerge from the synthesis. First, processing routes such as solution casting, spin coating, electrodeposition, electrospinning, sol–gel processing, chemical doping, and plasticization strongly influence crystallinity, amorphous fraction, morphology, filler dispersion, and interfacial organization. Second, in polymer electrolytes and dielectric systems, conductivity and relaxation behavior are mainly governed by salt dissociation, segmental mobility, electrode polarization, ion aggregation, and equivalent-circuit response. Third, optoelectronic and dielectric properties can be substantially tuned through metal oxides, MOFs, rare-earth oxides, quantum dots, and conductive additives, which introduce localized states, interfacial polarization, and modified optical transitions. Fourth, in electrochemical, impedance-sensing, and smart-device applications, chitosan functions as an interfacial material that supports immobilization, signal amplification, analyte interaction, and responsive charge-transfer behavior.

This review also shows that chitosan-based electronics should not be evaluated only through single performance indicators such as conductivity, band gap, dielectric constant, or detection limit. These properties are useful, but they need to be interpreted together with film thickness, humidity, stability, mechanical integrity, filler loading, transparency, reproducibility, real-sample validation, and sustainability considerations. Many studies report promising functional improvements, but cross-study comparison remains limited by inconsistent reporting of chitosan source, molecular weight, degree of deacetylation, processing conditions, environmental control, and long-term device stability.

To translate these findings into clearer strategic guidance, Table 7 organizes the priority research actions identified throughout this review into a short-term (1–3 years), medium-term (3–5 years), and long-term (5+ years) roadmap, linking each horizon to the enabling evidence already available in the reviewed corpus and to the specific gap that action is intended to close.

Overall, chitosan-based films and nanocomposites offer a credible pathway toward low-cost, flexible, biodegradable, and chemically tunable materials for green electronic applications. Their practical development, however, requires more standardized reporting, stronger device-level validation, and more careful assessment of sustainability at the full material-system level. Future studies should therefore combine molecular-level material design, controlled filler dispersion, reproducible processing, robust impedance and dielectric analysis, real-environment testing, and life-cycle-oriented evaluation. Such an integrated approach is needed for chitosan-based functional materials to move beyond laboratory-scale demonstrations and toward scalable, reliable, and environmentally responsible electronic technologies.

## Figures and Tables

**Table 1:** Representative summary of studies on structural engineering and processing routes of chitosan-based functional films.

Reference	Chitosan-Based System	Fabrication Method	Structural Modification Strategy	Characterization Methods	Main Structural Finding	Relevance to Sustainable Electronics
[9]	Chitosan:dextran solid polymer electrolyte with NH_4_CF_3_SO_3_	Solution casting	Proton-provider salt inserted into a CS:DX blend	FTIR, XRD, EIS, dielectric analysis	Salt dissociation and polymer–salt complexation altered crystallinity and enhanced ion transport; the highest DC conductivity reached 6.24 × 10^−6^ S/cm	Biodegradable polymer–electrolyte architecture for batteries, EDLCs, sensors, actuators, fuel cells, and solar-cell-related devices
[29]	Au/chitosan/n-InP hybrid junction	Spin coating	Chitosan used as a thin interfacial passivation layer	I–V, C–V, UV–Vis	The chitosan layer reduced reverse leakage current by approximately three orders of magnitude and increased the barrier height; the chitosan band gap was 4.60 eV	Chitosan as an active interfacial electronic modifier in hybrid junction devices
[46]	PPy–chitosan–Fe_3_O_4_ nanocomposite film on ITO	One-step electrochemical deposition	Conductive polymer and magnetic oxide incorporated into a chitosan film	FESEM, EDX, XRD, XPS, amperometry	Fe_3_O_4_ was incorporated into PPy–CS films; the electrode showed glucose linearity of 1–16 mM and an LOD of 234 μM	Electrodeposited chitosan nanocomposite films as biosensor coatings
[25]	CS:POZ/KI/glycerol plasticized polymer electrolyte	Solution casting	Glycerol plasticization of a KI-doped chitosan blend	EIS, EEC fitting, dielectric analysis, modulus analysis	Glycerol increased salt dissociation, mobile charge density, and ion transport; the highest conductivity reached 3.96 × 10^−4^ S/cm	Flexible electrolyte design for flexible electronics
[26]	CS:potato starch/KSCN biodegradable electrolyte	Solution casting	KSCN salt added as an ionic provider	FTIR, XRD, optical microscopy, EIS, EEC modeling	KSCN reduced the crystalline phase; FTIR deconvolution identified free ions, contact ion pairs, and ion aggregates	Structural disorder and ion speciation govern biodegradable electrolyte performance
[47]	Functionalized chitosan nanofiber membrane with TiO_2_	Electrospinning, chemical functionalization, electrospraying	TiO_2_ nanoparticles embedded in or surface-coated onto functionalized chitosan nanofibers	FTIR, XPS, SEM, TEM, XRD, EDX, BET	Functionalization changed nanofiber morphology, and TiO_2_ coating improved surface activity and photocatalytic function	Scalable nanofiber-processing route for functional green membranes
[15]	Ga_2_O_3_–CS and Pr_6_O_11_/Ga_2_O_3_–CS films	Solution casting with ultrasonication	Rare-earth oxide co-doping of chitosan films	XRD, SEM–EDS, FTIR, TGA, UV–Vis, impedance/modulus analysis	Crystallite size decreased from approximately 156 nm to 16 nm; doped films showed improved thermal, optical, and dielectric performance	Solvent-based route to chitosan dielectric films for electrical applications
[12]	CS/CMC/ZnO–Al_2_O_3_ nanocomposite films	Solution casting	ZnO nanorods and Al_2_O_3_ nanoparticles added to a CS/CMC blend	SEM, XRD, FTIR, UV–Vis, dielectric analysis, Nyquist plots, mechanical analysis	Nanofillers reduced crystallinity, enhanced optical absorption, and improved dielectric behavior	Flexible dielectric capacitors, optoelectronic films, and energy-storage substrates
[7]	P3HT-coated chitosan films	Spin coating and chemical doping	Conductive P3HT coating deposited onto chitosan and then doped with HAuCl_4_	ATR, UV–Vis, morphology, wettability, mechanical testing, conductivity testing	P3HT coating transformed chitosan into a conductive and flexible bio-based film	Chitosan as a platform for bio-based flexible electronics

Note: CS, chitosan; CMC, carboxymethyl cellulose; DX, dextran; EDLC, electric double-layer capacitor; EEC, electrical equivalent circuit; EIS, electrochemical impedance spectroscopy; FESEM, field-emission scanning electron microscopy; FTIR, Fourier-transform infrared spectroscopy; HRP, horseradish peroxidase; ITO, indium tin oxide; KSCN, potassium thiocyanate; MBTH, 3-methyl-2-benzothiazolinone hydrazone; P3HT, poly(3-hexylthiophene); POZ, poly(2-ethyl-2-oxazoline); PPy, polypyrrole; UV–Vis, ultraviolet–visible spectroscopy; XPS, X-ray photoelectron spectroscopy; XRD, X-ray diffraction.

**Table 5:** Benchmark comparison of best-reported performance metrics across the four dominant chitosan modification strategies identified in this review.

Modification Strategy	Representative System(s)	Highest Ionic/Electrical Conductivity	Peak Dielectric Constant (ε′)	Band-Gap Tuning Range	Lowest Reported LOD
Salt-doped and plasticized polymer electrolytes	Glutaraldehyde-crosslinked PVA–CS AEM [51]; CS/LiClO_4_ blend [50]; glycerol-plasticized CS-starch/NH_4_SCN [10]	24.6 × 10^−3^ S/cm, the highest value reported in this corpus [51]; range 6.24 × 10^−6^–24.6 × 10^−3^ S/cm across doped/plasticized systems [9,30,51]	Not the primary reported metric for this strategy	Not applicable (non-optoelectronic systems)	Not applicable
Metal-oxide and rare-earth-oxide nanocomposites	α-Fe_2_O_3_/PVA–CS [14]; ZrO_2_–CS [13]; TiO_2_/Fe_2_O_3_–CS [42]	Up to 5.1 × 10^−9^ S/cm DC conductivity, a dielectric-film regime not directly comparable to ionic-electrolyte conductivity [13]	2656 at 10 Hz, the highest value reported in this corpus [14]	Largest indirect-gap reduction: 4.76 → 1.57 eV [42]; largest direct-gap reduction: 5.76 → 4.68 eV [14]; 5.4 → 2.9 eV [13]	Not applicable
MOF-based composites	FeMOF-doped chitosan [17]; CrMOF-integrated chitosan [16]	Not reported as a conductivity value in either study	Reported only qualitatively as “dielectric modification”, not quantified numerically [16]	Largest band-gap reduction of any strategy in this review: 5.5 → 2.0 eV [17]; 5.5 → ~3 eV [16]	Not applicable
Carbon-based nanomaterials (graphene, MWCNT, graphite) and conductive-polymer composites	Au nanoparticles/graphene–chitosan immunosensor [58]; MWCNT–chitosan–Co [66]; graphite-reinforced NR/chitosan [68]	Percolation-threshold-dependent, approximately 30 wt% PANI [18]; conductivity increase of roughly six orders of magnitude over the unfilled polymer [68]	Not the primary reported metric for this strategy	Not applicable	0.7 fM (0.11 pg/mL), botulinum neurotoxin A [58]; 0.01 µmol/L, paracetamol [66]

Note: AEM, anion-exchange membrane; LOD, limit of detection; MOF, metal–organic framework; MWCNT, multi-walled carbon nanotube; NR, natural rubber; PANI, polyaniline. Values are the single best-case results reported in the primary studies summarized in Table 1, Table 2, Table 3 and Table 4 and Section 3.9 and are not directly comparable across strategies because they were measured under different protocols, film geometries, and environmental conditions (Section 3.8 discusses measurement-artifact and comparability limitations in more detail); “not applicable” indicates that the metric falls outside the primary performance domain targeted by that modification strategy, and “not reported” indicates that the underlying study did not quantify that metric numerically.

**Table 7:** Structured research roadmap for chitosan-based functional electronics, organized by time horizon.

Time Horizon	Priority Research Actions	Representative Enabling Evidence from This Review	Target Outcome/Rationale
Short-term (1–3 years)	Adopt standardized reporting of chitosan source, molecular weight, degree of deacetylation, film thickness, and relative humidity during electrical/dielectric testing; report electric-modulus-deconvolved dielectric data rather than raw apparent ε′	Modulus-based relaxation analysis already used in [30,48,53]; comparability gaps identified across Table 1, Table 2, Table 3, Table 4 and Table 5	Removes the measurement-artifact and reporting-inconsistency barriers identified in Section 3.8, enabling direct cross-study comparison
Medium-term (3–5 years)	Validate humidity-protective strategies (hydrophobic coating, fluorination, thin encapsulation) under controlled cycling; benchmark chitosan-based systems directly against established synthetic materials under matched test conditions; move MOF/metal-oxide systems from film-level to device-level demonstrations	Crosslinking-based humidity stabilization identified in [70,71]; benchmark values compiled in Table 5	Moves the field from isolated laboratory demonstrations toward device-relevant, comparable performance data
Long-term (5+ years)	Demonstrate intrinsically conductive, stretchable/wearable, and self-healing chitosan-based electronic systems validated under realistic operating conditions; integrate machine-learning-assisted composition-processing design; conduct full life-cycle/end-of-life assessment at the complete material-system level	Percolation-based conductive networks [18,68,74]; strain-tolerant hydrogel and ink systems [76,77,78]; ML-assisted electrochemical optimization [63]	Closes the coverage gaps identified in Section 3.9 (Table 6 extension) and completes the sustainability-and-translation feedback loop shown in Figure 4

Note: ML, machine learning; MOF, metal–organic framework. Entries are drawn exclusively from the primary studies and structural arguments already presented in this review; no additional literature search was conducted to populate this table.

## Data Availability

No new experimental data were generated in this study. The literature search strategy, eligibility criteria, screening procedure, and synthesized evidence are described in this article and the Supplementary Materials.

## Supplementary Materials

The following supporting information can be downloaded at https://www.mdpi.com/article/10.3390/ijms27177942/s1.
