# Supplementary material for: Chitosan-Based Functional Films and Nanocomposites for Sustainable Electronics: A Structure–Property–Function Systematic Review
Source: Int J Mol Sci. 2026 Sep 6;27(17):7942. doi: 10.3390/ijms27177942 (PMC13566041; doi:10.3390/ijms27177942)
Supplement: Supplementary file 1 [file ijms-27-07942-s001.zip › PRISMA_2020_checklist_completed_ijms-4552401.pdf]

PRISMA

# PRISMA 2020 Checklist

| Section and Topic | Item # | Checklist item | Location where item is reported |
|---|---|---|---|
| **TITLE** | | | |
| Title | 1 | Identify the report as a systematic review. | Title page: manuscript title identifies the report as “A Systematic Review”. |
| **ABSTRACT** | | | |
| Abstract | 2 | See the PRISMA 2020 for Abstracts checklist. | Abstract: summary includes background, aim, methods, thematic synthesis, main findings, limitations, and implications. |
| **INTRODUCTION** | | | |
| Rationale | 3 | Describe the rationale for the review in the context of existing knowledge. | Section 1. Introduction: rationale for chitosan-based sustainable electronics and the need for systematic structure–property–function synthesis. |
| Objectives | 4 | Provide an explicit statement of the objective(s) or question(s) the review addresses. | Section 1. Introduction: four review objectives stated in the final paragraph. |
| **METHODS** | | | |
| Eligibility criteria | 5 | Specify the inclusion and exclusion criteria for the review and how studies were grouped for the syntheses. | Section 2.1: inclusion/exclusion criteria (eligible chitosan/chitin-based systems reporting an electronic, optical, dielectric, impedance, electrochemical, or sensing property); Section 2.2: final 156 studies (176 initially retained, 20 removed on a subsequent bibliographic data-quality check) organized into four thematic synthesis groups. |
| Information sources | 6 | Specify all databases, registers, websites, organisations, reference lists and other sources searched or consulted to identify studies. Specify the date when each source was last searched or consulted. | Section 2.1: Scopus specified as the database; search performed 17–26 April 2026; additional focused searches described. |
| Search strategy | 7 | Present the full search strategies for all databases, registers and websites, including any filters and limits used. | Section 2.1, and Supplementary Table S1 and Figure S1: full Boolean query, five conceptual keyword blocks, filters, limits, and search logic; Supplementary Table S1 additionally reports four scoping queries used to position the review. |
| Selection process | 8 | Specify the methods used to decide whether a study met the inclusion criteria of the review, including how many reviewers screened each record and each report retrieved, whether they worked independently, and if applicable, details of automation tools used in the process. | Section 2.2 and Figure 1: PRISMA-based identification, duplicate removal, title/abstract screening, full-text eligibility assessment, and final inclusion, with the numeric breakdown of each stage. |
| Data collection process | 9 | Specify the methods used to collect data from reports, including how many reviewers collected data from each report, whether they worked independently, any processes for obtaining or confirming data from study investigators, and if applicable, details of automation tools used in the process. | Section 2.3: structured extraction matrix and extraction variables described. |
| Data items | 10a | List and define all outcomes for which data were sought. Specify whether all results that were compatible with each outcome domain in each study were sought (e.g. for all measures, time points, analyses), and if not, the methods used to decide which results to collect. | Section 2.3: outcomes sought included conductivity, dielectric constant, band gap, refractive index, ionic transference number, electrochemical stability, response/recovery time, limit of detection, linear range, sensitivity, and stability. |
| | 10b | List and define all other variables for which data were sought (e.g. participant and intervention characteristics, funding sources). Describe any assumptions made about any missing or unclear information. | Section 2.3: bibliographic data, chitosan form, fabrication method, dopant/filler/plasticizer, structural characterization, target device, sustainability claims, and limitations extracted; missing/unclear reporting treated as a limitation. |
| Study risk of bias assessment | 11 | Specify the methods used to assess risk of bias in the included studies, including details of the tool(s) used, how many reviewers assessed each study and whether they worked independently, and if applicable, details of automation tools used in the process. | Section 2.2 (final paragraph): quality assessed using materials-science criteria — composition clarity, synthesis procedure, characterization adequacy, measurement reliability, reproducibility indicators, and sustainability claims — with particular attention to impedance-spectroscopy interpretation. |
| Effect measures | 12 | Specify for each outcome the effect measure(s) (e.g. risk ratio, mean difference) used in the synthesis or presentation of results. | Not applicable: qualitative/thematic synthesis only; no pooled effect estimates. |

# PRISMA 2020 Checklist

| Section and Topic | Item # | Checklist item | Location where item is reported |
|---|---|---|---|
| Synthesis methods | 13a | Describe the processes used to decide which studies were eligible for each synthesis (e.g. tabulating the study intervention characteristics and comparing against the planned groups for each synthesis (item #5)). | Section 2.2: studies allocated to four thematic groups (structural engineering; dielectric/electrical/impedance; optoelectronic/band-gap; electrochemical/impedance-sensing), reflected in the organization of Sections 3.4–3.7. |
| | 13b | Describe any methods required to prepare the data for presentation or synthesis, such as handling of missing summary statistics, or data conversions. | Not applicable: no quantitative meta-analysis; no data conversion or imputation of missing summary statistics. |
| | 13c | Describe any methods used to tabulate or visually display results of individual studies and syntheses. | Section 2.3; Tables 1–4; Figures 2–4: tabular summaries and conceptual figures display individual-study and synthesis results. |
| | 13d | Describe any methods used to synthesize results and provide a rationale for the choice(s). If meta-analysis was performed, describe the model(s), method(s) to identify the presence and extent of statistical heterogeneity, and software package(s) used. | Section 2 (opening paragraph): thematic synthesis selected because the evidence was heterogeneous and unsuitable for meta-analysis. |
| | 13e | Describe any methods used to explore possible causes of heterogeneity among study results (e.g. subgroup analysis, meta-regression). | Not applicable: no subgroup analysis or meta-regression because no meta-analysis was performed. |
| | 13f | Describe any sensitivity analyses conducted to assess robustness of the synthesized results. | Not applicable: no statistical sensitivity analysis because no statistical synthesis was performed. |
| Reporting bias assessment | 14 | Describe any methods used to assess risk of bias due to missing results in a synthesis (arising from reporting biases). | Not applicable/not formally assessed: no meta-analysis or outcome-level reporting-bias assessment; reporting limitations discussed qualitatively. |
| Certainty assessment | 15 | Describe any methods used to assess certainty (or confidence) in the body of evidence for an outcome. | Section 2.2 (quality-assessment paragraph) and Section 3.9: confidence addressed qualitatively through methodological-quality and reporting-completeness appraisal rather than GRADE. |
| **RESULTS** | | | |
| Study selection | 16a | Describe the results of the search and selection process, from the number of records identified in the search to the number of studies included in the review, ideally using a flow diagram. | Section 2.2 and Figure 1: records identified, filtered, duplicates removed, screened, full texts assessed, excluded with reasons, and final included studies reported. |
| | 16b | Cite studies that might appear to meet the inclusion criteria, but which were excluded, and explain why they were excluded. | Section 2.2: full-text exclusion reasons reported by category; individual excluded-study citations were not listed. |
| Study characteristics | 17 | Cite each included study and present its characteristics. | Sections 3.4–3.7 and Tables 1–4: included studies cited and summarized by system, fabrication/modification, characterization, performance, and application relevance. |
| Risk of bias in studies | 18 | Present assessments of risk of bias for each included study. | Section 2.2 (quality-assessment paragraph) and Section 3.9: methodological/reporting limitations discussed qualitatively; no study-level risk-of-bias table used because evidence consisted mainly of heterogeneous materials/device studies. |
| Results of individual studies | 19 | For all outcomes, present, for each study: (a) summary statistics for each group (where appropriate) and (b) an effect estimate and its precision (e.g. confidence/credible interval), ideally using structured tables or plots. | Sections 3.4–3.7 and Tables 1–4: individual-study findings and quantitative performance metrics presented where applicable. |
| Results of syntheses | 20a | For each synthesis, briefly summarise the characteristics and risk of bias among contributing studies. | Sections 3.4–3.7 and Tables 1–4: characteristics and methodological/reporting limitations summarized within each thematic synthesis. |
| | 20b | Present results of all statistical syntheses conducted. If meta-analysis was done, present for each the summary estimate and its precision (e.g. confidence/credible interval) and measures of statistical heterogeneity. If comparing groups, describe the direction of the effect. | Not applicable: no statistical synthesis or meta-analysis conducted. |
| | 20c | Present results of all investigations of possible causes of heterogeneity among study results. | Not applicable: heterogeneity discussed narratively in Section 3.9; no subgroup analysis/meta-regression. |
| | 20d | Present results of all sensitivity analyses conducted to assess the robustness of the | Not applicable: no sensitivity analysis conducted. |

PRISMA

# PRISMA 2020 Checklist

| Section and Topic | Item # | Checklist item | Location where item is reported |
|---|---|---|---|
| | | synthesized results. | |
| Reporting biases | 21 | Present assessments of risk of bias due to missing results (arising from reporting biases) for each synthesis assessed. | Not applicable/not formally assessed: missing-result/reporting-bias assessment not performed; reporting gaps discussed qualitatively in Section 3.9. |
| Certainty of evidence | 22 | Present assessments of certainty (or confidence) in the body of evidence for each outcome assessed. | Section 2.2 and Section 3.9: certainty/confidence considered qualitatively through reproducibility, reporting completeness, environmental control, and device-validation limitations. |
| **DISCUSSION** | | | |
| Discussion | 23a | Provide a general interpretation of the results in the context of other evidence. | Section 3.9 and Section 4 (Conclusion): interpretation of results in the context of chitosan-based sustainable electronics. |
| | 23b | Discuss any limitations of the evidence included in the review. | Section 3.9 and Section 4 (Conclusion, third paragraph): limitations of included evidence, including inconsistent reporting of chitosan source, molecular weight, degree of deacetylation, film thickness, humidity, stability, and sustainability metrics. |
| | 23c | Discuss any limitations of the review processes used. | Section 2.2 (quality-assessment paragraph) and Section 3.9: limitations of the review process and cross-study comparability discussed, including heterogeneity and the absence of meta-analysis. |
| | 23d | Discuss implications of the results for practice, policy, and future research. | Section 3.9 and Section 4 (Conclusion): implications for reporting standards, scalable processing, device validation, sustainability assessment, and future research. |
| **OTHER INFORMATION** | | | |
| Registration and protocol | 24a | Provide registration information for the review, including register name and registration number, or state that the review was not registered. | Section 2 Methods: review protocol was not prospectively registered; no registration number available. |
| | 24b | Indicate where the review protocol can be accessed, or state that a protocol was not prepared. | Section 2 Methods: no publicly accessible registered protocol was prepared. |
| | 24c | Describe and explain any amendments to information provided at registration or in the protocol. | Not applicable: review was not registered and no registered protocol amendments were made. |
| Support | 25 | Describe sources of financial or non-financial support for the review, and the role of the funders or sponsors in the review. | Funding statement: Universitas Terbuka funding and contract number reported. |
| Competing interests | 26 | Declare any competing interests of review authors. | Conflicts of Interest statement: authors declare no conflicts of interest. |
| Availability of data, code and other materials | 27 | Report which of the following are publicly available and where they can be found: template data collection forms; data extracted from included studies; data used for all analyses; analytic code; any other materials used in the review. | Data Availability Statement and Supplementary Materials statement: no new experimental data generated; search strategy, eligibility criteria, screening procedure, and synthesized evidence described in the article; Figure S1 (Boolean search-strategy map), Table S1 (search-strategy/yield data), and Table S2 (full list of the 156 included studies) provided as Supplementary Materials; this completed PRISMA 2020 checklist provided as an additional supplementary file. |

*From:* Page MJ, McKenzie JE, Bossuyt PM, Boutron I, Hoffmann TC, Mulrow CD, et al. The PRISMA 2020 statement: an updated guideline for reporting systematic reviews. BMJ 2021;372:n71. doi: 10.1136/bmj.n71. This work is licensed under CC BY 4.0. To view a copy of this license, visit https://creativecommons.org/licenses/by/4.0/
