# Supplementary material for: Chitosan-Based Functional Films and Nanocomposites for Sustainable Electronics: A Structure–Property–Function Systematic Review
Source: Int J Mol Sci. 2026 Sep 6;27(17):7942. doi: 10.3390/ijms27177942 (PMC13566041; doi:10.3390/ijms27177942)
Supplement: Supplementary file 1 [file ijms-27-07942-s001.zip › Supplementary_Materials.pdf]

Supplementary Materials

Chitosan-Based Functional Films and Nanocomposites for Sustainable Electronics: A Structure–Property–Function Systematic Review

Figure S1. Boolean Search Strategy Map

The search logic was organized into five conceptual blocks — material, structure/nanocomposite, property/performance, function/application, and processing/modification — combined with Boolean AND/OR operators as described in Section 2.1 of the main text. The original search-strategy figure (previously submitted as Figure S1) is retained unchanged.

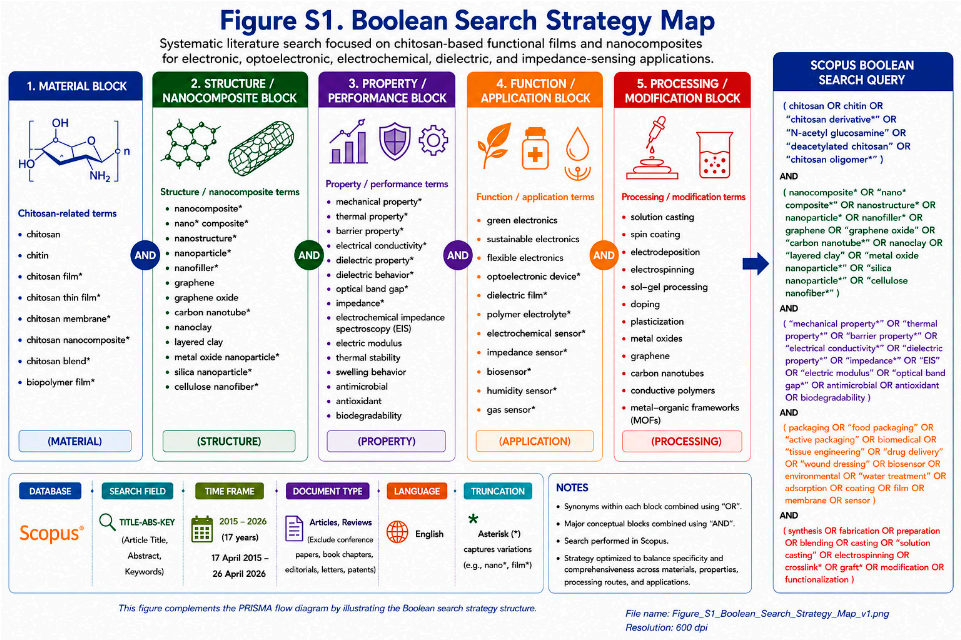

Table S1. Search strategy and preliminary Scopus yield used to position the review scope

(Reproduced from the original submission; unchanged.)

Search focus	Search string summary	Initial records	Filtered records (2015-2026; article/review; English; journal)
Broad chitosan electronics	chitosan OR chitin; thin film/membrane/coating/biopolymer; electronic/dielectric/optoelectronic/sensor	3,764	3,255
Green electronics focus	chitosan OR chitin; thin film/coating; green/sustainable electronics OR biodegradable/bioelectronic; dielectric/conductivity/optoelectronic/sensor	129	104
Structure-property focus	chitosan; thin film/membrane; structure-property/morphology/crystallinity/DD; dielectric/conductivity/impedance/band gap	280	237
Waste-origin focus	chitosan; shrimp shell/crustacean waste/shell waste/marine waste/seafood waste; thin film/membrane/coating; dielectric/optoelectronic/impedance/sensor	11	9
Impedance/optoelectronic focus	chitosan; thin film/coating; impedance/humidity/dielectric/electrical conductivity/optical band gap/optoelectronic; morphology/crystallinity/DD/microstructure	162	135

Table S2. Full list of studies included in the qualitative synthesis (n = 156)

This table lists every study included in the final qualitative synthesis reported in Section 2.3 and Figure 1 (PRISMA flow diagram) of the main text. The reference numbers in the "Ref" column below are internal to this table and are independent of the main-text reference list, which (per standard journal practice) contains only the works actually cited by number in the main-text narrative. Of the 156 studies listed here, 54 are also cited individually in the main-text narrative and appear in Tables 1-4 or the accompanying discussion (Sections 3.1-3.9); these are marked "Main-text" in the Source column. The remaining 102 studies were identified from the same literature-search corpus, screened against the eligibility criteria in Section 2.2, and are documented here with full bibliographic detail and thematic classification even though they are not individually named in the main-text discussion; they are instead reflected there through corpus-level statements (e.g., study counts by theme) and the Section 3.9 emerging-topics analysis. This total reflects a bibliographic data-quality check that removed 20 studies falling outside the review's stated inclusion window (16 published before 2015; 4 published after the 26 April 2026 Scopus search date) from an initially retained set of 176. It also resolves the discrepancy between the number of studies reported as included (previously stated as 168) and the number of works appearing in the reference list, raised in the first round of review, and reflects the reference-list consolidation completed in response to the editor comments in the current (minor) revision round.

Ref	Citation	Theme	Source	Emerging-topic flag
25	Abdullah, R.M. Investigation of Ion Transport in Plasticized Polymer Electrolytes Using Electrical Equivalent Circuit (EEC) Modeling. *J Mater Sci Mater Electron* **2024**, *35*, 777, doi:10.1007/s10854-024-12564-x.	(see Tables 1-4)	Main-text	-
26	Abdulwahid, R.T.; Aziz, S.B.; Kadir, M.F.Z. Insights into Ion Transport in Biodegradable Solid Polymer Blend Electrolyte Based on FTIR Analysis and Circuit Design. *J Phys Chem Solids* **2022**, *167*, 110774, doi:10.1016/j.jpcs.2022.110774.	(see Tables 1-4)	Main-text	-
27	da Silva, G.M.G.; Faia, P.M.; Mendes, S.R.; Araújo, E.S. A Review of Impedance Spectroscopy Technique: Applications, Modelling, and Case Study of Relative Humidity Sensors Development. *Applied Sciences* **2024**, *14*, 5754, doi:10.3390/app14135754.	(see Tables 1-4)	Main-text	-
29	Alghunaim, N.S. Effect of Blending on the Enhancement of Electronic Properties of Biopolymers. *J. Comput. Theor. Nanosci.* **2016**, *13*, 6800--6802, doi:10.1166/jctn.2016.5630.	(see Tables 1-4)	Main-text	-
30	Abay, B. Barrier Characteristics of Biopolymer-Based Organic/Inorganic Au/CTS/n-InP Hybrid Junctions. *Philos. Mag.* **2015**, *95*, 3413--3428, doi:10.1080/14786435.2015.1076583.	(see Tables 1-4)	Main-text	-
31	Abdullah, O.G.; Hanna, R.R.; Ahmed, H.T.; Mohamad, A.H.; Saleem, S.A.; Saeed, M.A.M. Conductivity and Dielectric Properties of Lithium-Ion Biopolymer Blend Electrolyte Based Film. *Results Phys.* **2021**, *24*, 104135, doi:10.1016/j.rinp.2021.104135.	(see Tables 1-4)	Main-text	-
32	Alanazi, T.I.; El Sayed, A.M. M^{3+}/$NaTiO_3$/PVA-Chitosan Nanocomposites (M = Ga, Ce, Nd or Er): Novel Solid Polymer Electrolytes for Supercapacitors. *Phys Scr* **2023**, *98*, 085946, doi:10.1088/1402-4896/ace85d.	(see Tables 1-4)	Main-text	-
33	Alsalmah, H.A.; Rajeh, A.; Farea, M.O.; Nur, O. Synthesis and Characterization of PEG/CS-$AgNO_3$ Polymer Nanocomposites for Flexible Optoelectronic and Energy Storage Applications. *Polym Compos* **2024**, *45*, 5164--5175, doi:10.1002/pc.28118.	(see Tables 1-4)	Main-text	-
34	Nafati, H.; Litaiem, Y.; Bouya Ahmed, I.; Choubani, K.; Ballarin, B.; Almeshaal, M.A.; Rabha, M.B.; Dimassi, W. High-Performance Flexible Nanocomposite Networks Based on Grafted Chitosan--PANI for Flexible Electronics. *Crystals* **2026**, *16*, 255, doi:10.3390/cryst16040255.	(see Tables 1-4)	Main-text	-
35	Nagaraja, M.; Prashanth, S.; Mokshanatha, P.B.; Pattar, J.; Manohara, S.R.; Sunil, K. Tuning Electrical Conductivity and Dielectric Properties of Chitosan/Polyaniline by Doping with Varying Concentration of Nb_2O_5 Nanoparticles. *Solid State Ionics* **2026**, *437*, 117129, doi:10.1016/j.ssi.2026.117129.	(see Tables 1-4)	Main-text	-
36	Prokhorov, Y.; Luna-Barcenas, G.; Kovalenko, Y. Interphase Percolation Phenomena in Chitosan-Graphene Oxide Nanocomposites, the Role of Water Content. *Express Polym. Lett.* **2023**, *17*, 29--39, doi:10.3144/expresspolymlett.2023.3.	(see Tables 1-4)	Main-text	-
37	Somanathan, S.; Aswal, V.K.; Ramasamy, R.P. Broadband Dielectric Spectroscopy and Small-Angle Neutron Scattering Investigations of Chitosan-	(see Tables 1-4)	Main-text	-

Ref	Citation	Theme	Source	Emerging-topic flag
	-Graphene--Silver Metacomposites. *J Mater Sci Mater Electron* **2022**, *33*, 217--234, doi:10.1007/s10854-021-07287-2.			
38	Begum, S.N.S.; Aswal, V.K.; Ramasamy, R.P. Small-Angle Neutron Scattering and Spectroscopic Investigations of Ag Fractal Formation in Chitosan-Ag Nanocomposite Facilitated by Hydrazine Hydrate. *J. Phys. Chem. C* **2016**, *120*, 2400--2410, doi:10.1021/acs.jpcc.5b09747.	(see Tables 1-4)	Main-text	-
39	Meera, K.; Ramesan, M.T. Development of High-Performance Biopolymer Nanocomposites Derived from Carboxymethyl Chitosan/Boehmite via Green Synthesis. *Polym Compos* **2023**, *44*, 1135--1148, doi:10.1002/pc.27159.	(see Tables 1-4)	Main-text	-
40	Meera, K.; Arun, K.; Ramesan, M.T. Nanochitosan Reinforced Polyvinyl Alcohol/Cashew Gum Bio-Blend Nanocomposites: Promising Materials for Future Frontiers. *J. Polym. Environ.* **2023**, *31*, 4487--4505, doi:10.1007/s10924-023-02909-8.	(see Tables 1-4)	Main-text	-
41	Pinto, J.P.; D'souza, O.J.; Chavan, C.; Bhajanthri, R.F.; Masti, S.P.; Chougale, R.B. Tuning the Energy Storage Performance of Graphene Oxide-Based Nanocomposite Films by Encapsulating Silver Nanoparticles. *Diamond Relat. Mat.* **2023**, *140*, 110483, doi:10.1016/j.diamond.2023.110483.	(see Tables 1-4)	Main-text	-
42	Ramesan, M.T.; Sankar, S.; Kalladi, A.J.; Labeeba Abdulla, A.C.; Bahuleyan, B.K. Hydroxyapatite Nanoparticles Reinforced Polyvinyl Alcohol/Chitosan Blend for Optical and Energy Storage Applications. *Polym Eng Sci* **2024**, *64*, 1378--1390, doi:10.1002/pen.26623.	(see Tables 1-4)	Main-text	-
43	Domyati, D. Dielectric and Optoelectronics Properties of Thin Films Based on Chitosan Containing Titanium Oxide/Hematite and Their Potential for Optoelectronics Devices. *J Mater Sci Mater Electron* **2024**, *35*, 406, doi:10.1007/s10854-024-12102-9.	(see Tables 1-4)	Main-text	-
45	Ragab, H.M.; Diab, N.S.; Khaled, A.M.; Al Ojeery, A.; Al-Hakimi, A.N.; Farea, M.O. Incorporating Hybrid Ag/Co_2O_3 Nanofillers into PVP/CS Blends for Multifunctional Optoelectronic and Nanodielectric Applications. *Ceram Int* **2024**, *50*, 1254--1262, doi:10.1016/j.ceramint.2023.10.219.	(see Tables 1-4)	Main-text	-
46	El Sayed, A.M.; El-Gamal, S. Synthesis, Optical, and Electrical Properties of Starch/Chitosan/$NaTiO_3$bio-Nanocomposites Modified with $ErCl_3$. *Phys Scr* **2022**, *97*, 015805, doi:10.1088/1402-4896/ac40da.	(see Tables 1-4)	Main-text	-
47	Ezzat, H.A.; Hegazy, M.A.; Nada, N.A.; Osman, O.; Ibrahim, M.A. Studying the Optical and Thermal Properties of Cs/ZnO and Cs/ZnO/GO Hybrid Nanocomposites. *Opt Mater* **2023**, *135*, 113244, doi:10.1016/j.optmat.2022.113244.	(see Tables 1-4)	Main-text	-
48	Abdul Amir AL-Mokaram, A.M.A.; Yahya, R.; Abdi, M.M.; Muhammad Ekramul Mahmud, H.N. One-Step Electrochemical Deposition of Polypyrrole--Chitosan--Iron Oxide Nanocomposite Films for Non-Enzymatic Glucose Biosensor. *Mater Lett* **2016**, *183*, 90--93, doi:10.1016/j.matlet.2016.07.049.	(see Tables 1-4)	Main-text	-
50	AlAbduljabbar, F.A.; Haider, S.; Ahmed Ali, F.A.; Alghyamah, A.A.; Almasry, W.A.; Patel, R.; Mujtaba, I.M. TiO_2 Nanostructured Coated Functionally Modified and Composite Electrospun Chitosan Nanofibers Membrane for Efficient Photocatalytic Degradation of Organic Pollutant in Wastewater. *J. Mater. Res. Technol.* **2021**, *15*, 5197--5212, doi:10.1016/j.jmrt.2021.10.119.	(see Tables 1-4)	Main-text	-
51	Abdulwahid, R.T.; Aziz, S.B.; Kadir, M.F.Z. Design of Proton Conducting Solid Biopolymer Blend Electrolytes Based on Chitosan-Potato Starch Biopolymers: Deep Approaches to Structural and Ion Relaxation Dynamics of H+ Ion. *J. Appl. Polym. Sci.* **2022**, *139*, e52892, doi:10.1002/app.52892.	(see Tables 1-4)	Main-text	-
52	Abdulwahid, R.T.; Aziz, S.B.; Kadir, M.F.Z.; Sadiq, N.M.; Halim, N.A.; Hamsan, M.H.; Saeed, S.R.; Woo, H.J. Biodegradable Polymer Membrane K+ Ion Conductor for Electrochemical Device Application. *J Mater Sci* **2022**, *57*, 19902--19923, doi:10.1007/s10853-022-07825-1.	(see Tables 1-4)	Main-text	-
53	Aziz, S.B.; Hamsan, M.H.; Kadir, M.F.Z.; Karim, W.O.; Abdullah, R.M. Development of Polymer Blend Electrolyte Membranes Based on Chitosan: Dextran with High Ion Transport Properties for EDLC Application. *Int. J. Mol. Sci.* **2019**, *20*, 3369, doi:10.3390/ijms20133369.	(see Tables 1-4)	Main-text	-

Ref	Citation	Theme	Source	Emerging-topic flag
54	Ahmed, S.M.; Rashid, K.T.; AbdulRazak, A.A.; Allami, S.; Alsalhy, Q.F. Eco-Friendly Synthesis of Alkaline Anion Exchange Membrane for Fuel Cells Application. *Brazil J Chem Eng* **2022**, *39*, 183--195, doi:10.1007/s43153-021-00189-0.	(see Tables 1-4)	Main-text	-
55	Aziz, S.B.; Aziz, D.M.; Hama, P.O.; Abdulwahid, R.T.; Sadiq, N.M.; Al-Saeedi, S.I.; Kadir, M.F.Z.; Sangaraju, S.; Yong, J.; Woo, H.J. Unlocking High Energy Density and Stable Electric Double Layer Capacitor Derived from Sustainable Biodegradable Polymer Electrolyte Based on Chitosan:Polyvinyl Alcohol: Potassium Thiocyanate:xGlycerol (10≤x≤30). *Result. Eng.* **2025**, *27*, 106897, doi:10.1016/j.rineng.2025.106897.	(see Tables 1-4)	Main-text	-
56	Aziz, S.B.; Ahmed, M.J.; Abdullah, O.G.; Murad, A.R.; Hamad, S.M.; Hadi, J.M. Magnesium Ion Conducting Biopolymer Blend-Based Electrolyte for Energy Storage Application: Electrochemical Characteristics. *Electrochim Acta* **2023**, *461*, 142659, doi:10.1016/j.electacta.2023.142659.	(see Tables 1-4)	Main-text	-
57	Ali, F.I.M.; Awwad, F.; Greish, Y.E.; Abu-Hani, A.F.S.; Mahmoud, S.T. Fabrication of Low Temperature and Fast Response H_2S Gas Sensor Based on Organic-Metal Oxide Hybrid Nanocomposite Membrane. *Org. Electron* **2020**, *76*, doi:10.1016/j.orgel.2019.105486.	(see Tables 1-4)	Main-text	-
58	Alghamdi, H.M.; Rajeh, A. Study of the Photoluminescence, Optical, Thermal, and Electrical Parameters of the Cs/PVP Blend/Zinc Oxide Nanorods Films for Energy Storage Devices. *Polym Test* **2023**, *124*, 108093, doi:10.1016/j.polymertesting.2023.108093.	(see Tables 1-4)	Main-text	-
59	Abdelghany, A.M.; Meikhail, M.S.; Awad, W.M. Electrical Conductivity of Chitosan/PCL Hosting Network for CdSe Quantum Dots. *Polym. Bull.* **2022**, *79*, 4381--4393, doi:10.1007/s00289-021-03715-9.	(see Tables 1-4)	Main-text	-
60	Al-Refai, H.; Bashal, A.H.; Khalil, K.D.; Zaki, A.A. Optical Enhancement of Chitosan Films Through Neodymium Oxide Nanoparticle Doping: Experimental and Molecular Dynamics Insights for Advanced Material Applications. *J. Inorg. Organomet. Polym. Mater.* **2025**, *35*, 3698--3709, doi:10.1007/s10904-024-03486-6.	(see Tables 1-4)	Main-text	-
61	Afkhami, A.; Hashemi, P.; Bagheri, H.; Salimian, J.; Ahmadi, A.; Madrakian, T. Impedimetric Immunosensor for the Label-Free and Direct Detection of Botulinum Neurotoxin Serotype A Using Au Nanoparticles/Graphene-Chitosan Composite. *Biosens. Bioelectron.* **2017**, *93*, 124--131, doi:10.1016/j.bios.2016.09.059.	(see Tables 1-4)	Main-text	-
62	Abd-Elsabour, M.; Abou-Krisha, M.M.; Abdel-Haleem, F.M.; Goda, M.N.; Mohy-Eldin, M.S. Electrochemical Nanomolar Determination of the Anticancer Drug 5-Fluorouracil Using Chitosan Curcumin Schiff Base-Decorated NiO Nanorods Synthesized with Green Method. *J Electrochem Soc* **2024**, *171*, 087520, doi:10.1149/1945-7111/ad7171.	(see Tables 1-4)	Main-text	-
63	Aghris, S.; Azriouil, M.; Matrouf, M.; Ettadili, F.E.; Laghrib, F.; Saqrane, S.; Farahi, A.; Bakasse, M.; Lahrich, S.; El Mhammedi, M.A. Chitosan Biopolymer Coated Graphite Electrode as a Robust Electrochemical Platform for the Detection of the Insecticide Flubendiamide. *J. Food Compos. Anal.* **2022**, *114*, doi:10.1016/j.jfca.2022.104749.	(see Tables 1-4)	Main-text	-
64	Saisa; Agusnar, H.; Alfian, Z.; Nainggolan, I. Chitosan-Zinc Oxide Nanoparticle Based Highly Sensitive Electrochemical Sensor for Formaldehyde Detection. *Rasayan J. Chem.* **2020**, *13*, 2530--2537, doi:10.31788/RJC.2020.1345786.	(see Tables 1-4)	Main-text	-
65	Akanbi, F.S.; Yusof, N.A.; Abdullah, J.; Sulaiman, Y.; Hushiarian, R. Detection of Quinoline in G. boninense-Infected Plants Using Functionalized Multi-Walled Carbon Nanotubes: A Field Study. *Sensors* **2017**, *17*, doi:10.3390/s17071538.	(see Tables 1-4)	Main-text	-
66	Ahmed, Y.; Akter, T.; Prima, M.; Dutta, K.R.; Mukut, S.; Ahsan, M.; Rahman, M.M.; Mohammad Ziaul Hyder, M.K. Advanced Ciprofloxacin Quantification: A Machine Learning and Metaheuristic Approach Using Ultrasensitive Chitosan-Gold Nanoparticle Based Electrochemical Sensor. *J. Environ. Chem. Eng.* **2025**, *13*, doi:10.1016/j.jece.2024.115094.	(see Tables 1-4)	Main-text	-
67	Abdollahi-Esfahlani, H.; Sabzmeydani, H.; Pourmahdian, S.; Tanha, A.; Hadjizadeh, A. Development of Three-Dimension Polyaniline-Based Ternary Hierarchical Architectures as Chemiresistive Ammonia Gas Sensor and Smart Green Label for Fish Freshness. *Microchem. J.* **2025**, *217*, doi:10.1016/j.microc.2025.114845.	(see Tables 1-4)	Main-text	-

Ref	Citation	Theme	Source	Emerging-topic flag
68	Adeosun, W.A.; Asiri, A.M.; Marwani, H.M. Fabrication of Conductive Polypyrrole Doped Chitosan Thin Film for Sensitive Detection of Sulfite in Real Food and Biological Samples. *Electroanalysis* **2020**, *32*, 1725--1736, doi:10.1002/elan.201900765.	(see Tables 1-4)	Main-text	-
69	Akhter, S.; Basirun, W.J.; Alias, Y.; Johan, M.R.; Bagheri, S.; Shalauddin, M.; Ladan, M.; Anuar, N.S. Enhanced Amperometric Detection of Paracetamol by Immobilized Cobalt Ion on Functionalized MWCNTs - Chitosan Thin Film. *Anal. Biochem.* **2018**, *551*, 29--36, doi:10.1016/j.ab.2018.05.004.	(see Tables 1-4)	Main-text	-
70	Shrestha, B.K.; Ahmad, R.; Mousa, H.M.; Kim, I.-G.; Kim, J.I.; Neupane, M.P.; Park, C.H.; Kim, C.S. High-Performance Glucose Biosensor Based on Chitosan-Glucose Oxidase Immobilized Polypyrrole/Nafion/Functionalized Multi-Walled Carbon Nanotubes Bio-Nanohybrid Film. *J. Colloid Interface Sci.* **2016**, *482*, 39--47, doi:10.1016/j.jcis.2016.07.067.	(see Tables 1-4)	Main-text	-
71	Hunais, O.M.; Ramseena, T.A.; Meera, K.; Bahuleyan, B.K.; Ramesan, M.T. Tuning the Structural, Mechanical, Thermal and Electrical Properties of in-Situ Polymerized Polyindole/Carboxymethyl Chitosan/Nickel Oxide Blend Nanocomposites for Energy Storage Applications. *Journal of Polymers and the Environment*, *32*(8), 4035--4045. 2024, doi:10.1007/s10924-024-03250-4.	Structural engineering & processing	S2 only	conductive
73	Kitsawat, V.; Siri, S.; Pakawanit, P.; Phisalaphong, M. Improved Electrical Conductivity and Mechanical Properties of Natural Rubber Composite Films by Incorporating Graphite and Chitosan. *J. Appl. Polym. Sci.* **2025**, *142*, doi:10.1002/app.57497.	Structural engineering & processing	Main-text	conductive
75	Sun, S.; Wang, Y.; Li, L.; Huang, Z.; Zhou, H. Thermoplastic biomass transparent films directly fabricated by chitosan nanospheres. *Polymer*, *192*. 2020, doi:10.1016/j.polymer.2020.122335.	Structural engineering & processing	S2 only	-
76	Ali, Q.; Taweepreda, W.; Techato, K. Preparation and Characterization of Polymer Electrolyte Membrane from Chloroacetate Chitosan/Chitosan Blended with Epoxidized Natural Rubber. *Polym. Test.* **2020**, *82*, 106294, doi:10.1016/j.polymertesting.2019.106294.	Dielectric/electrical properties & polymer electrolytes	Main-text	-
77	Asnawi, A.S.F.M.; Aziz, S.B.; Brevik, I.; Brza, M.A.; Yusof, Y.M.; Alshehri, S.M.; Ahamad, T.; Kadir, M.F.Z. The study of plasticized sodium ion conducting polymer blend electrolyte membranes based on chitosan/dextran biopolymers: Ion transport, structural, morphological and potential stability. *Polymers*, *13*(3), 1--25. 2021a, doi:10.3390/polym13030383.	Dielectric/electrical properties & polymer electrolytes	S2 only	-
78	Aziz, S.B.; Asnawi, A.S.F.M.; Mohammed, P.A.; Abdulwahid, R.T.; Yusof, Y.M.; Abdullah, R.M.; Kadir, M.F.Z. Impedance, circuit simulation, transport properties and energy storage behavior of plasticized lithium ion conducting chitosan based polymer electrolytes. *Polymer Testing*, *101*. 2021, doi:10.1016/j.polymertesting.2021.107286.	Dielectric/electrical properties & polymer electrolytes	S2 only	-
79	Aziz, S.B.; Abdulwahid, R.T.; Sadiq, N.M.; Abdullah, R.M.; Tahir, D.A.; Jameel, D.A.; Hamad, S.M.; Abdullah, O.G. Design of biodegradable polymer blend electrolytes with decoupled ion motion for EDLC device Application: Electrical and electrochemical properties. *Results in Physics*, *51*. 2023, doi:10.1016/j.rinp.2023.106692.	Dielectric/electrical properties & polymer electrolytes	S2 only	-
80	Aziz, M.F.; Shamsuri, N.A.; Hamsan, M.H.; Yusof, Y.M.; Azam, M.A.; Aziz, S.B.; Rusdi, H.; Kadir, M.F.Z.; Shukur, M.F. An elucidation of symmetric supercapacitor with crustacean-based polymer blend electrolyte. *Journal of Applied Polymer Science*, *141*(2). 2024, doi:10.1002/app.54781.	Dielectric/electrical properties & polymer electrolytes	S2 only	-
81	Aziz, D.M.; Abdulwahid, R.T.; Hassan, S.A.; Aziz, S.B.; Singh, P.K.; Al-Asbahi, B.A.; Ahmed, A.A.A.; Woo, H.J.; Kadir, M.F.Z.; Karim, W.O. Development and Investigation of Electrochemical and Dielectric Properties of Eco-Friendly Lithium-Ion Conductor Biopolymer Electrolyte for Energy Storage Application. *Journal of Polymers and the Environment*, *32*(8), 3845--3868. 2024, doi:10.1007/s10924-024-03198-5.	Dielectric/electrical properties & polymer electrolytes	S2 only	-
82	Aziz, S.B.; Hama, P.O.; Abdulwahid, R.T.; Aziz, D.M.; Mohammed, P.A.; Hamsan, M.H.; Abdullah, R.M.; Kadir, M.F.Z. Plasticized ecofriendly chitosan-potato starch biopolymer electrolyte with enhanced dielectric constant for EDLC application. *Emergent Materials*, *8*(4), 2933--2958. 2025, doi:10.1007/s42247-024-00981-x.	Dielectric/electrical properties & polymer electrolytes	S2 only	-
83	Babakr, K.A.; Qader, I.N.; Rasul, H.H.; Aziz, S.B.; Mohammed, S.S.; Hamid, D.A.; Salih, I.L.; Mahmood, P.H.; Ibrahim, P.A.; Muhammed, A.W.; Ali,	Dielectric/electrical properties &	S2 only	-

Ref	Citation	Theme	Source	Emerging-topic flag
	B.T.; Aspoukeh, P.; Khojasteh, H.; Hamad, S.M. Molecular-level insights into glycerol-assisted ion transport and dielectric behavior of CS--PVA--NaSCN--Al2O3 polymer electrolytes. *Journal of Materials Science*, *60*(48), 25516--25542. 2025a, doi:10.1007/s10853-025-11917-z.	polymer electrolytes		
85	Brza, M.; Aziz, S.B.; Saeed, S.R.; Hamsan, M.H.; Majid, S.R.; Abdulwahid, R.T.; Kadir, M.F.Z.; Abdullah, R.M. Energy storage behavior of lithium-ion conducting poly(Vinyl alcohol) (pva): Chitosan(cs)-based polymer blend electrolyte membranes: Preparation, equivalent circuit modeling, ion transport parameters, and dielectric properties. *Membranes*, *10*(12), 1--20. 2020, doi:10.3390/membranes10120381.	Dielectric/electrical properties & polymer electrolytes	S2 only	-
86	Bósquez-Cáceres, M.F.; Lima, L.D.; Morera Córdova, V.; Delgado, A.D.; Béjar, J.; Arjona, N.; Álvarez-Contreras, L.; Tafur, J.P. Chitosan-Carboxymethylcellulose Hydrogels as Electrolytes for Zinc--Air Batteries: An Approach to the Transition towards Renewable Energy Storage Devices. *Batteries*, *8*(12). 2022, doi:10.3390/batteries8120265.	Dielectric/electrical properties & polymer electrolytes	S2 only	-
87	Ehsani, A.; Parsimehr, H.; Nourmohammadi, H.; Safari, R.; Doostikhah, S. Environment-friendly electrodes using biopolymer chitosan/poly ortho aminophenol with enhanced electrochemical behavior for use in energy storage devices. *Polymer Composites*, *40*(12), 4629--4637. 2019a, doi:10.1002/pc.25330.	Dielectric/electrical properties & polymer electrolytes	S2 only	conductive
88	Erol, İ.; Khamidov, G. Enhanced dielectric and surface properties of chitosan-based nanocomposites reinforced with Ag-GO nanoparticles. *Polymer Bulletin*, *83*(2). 2026, doi:10.1007/s00289-025-06180-w.	Dielectric/electrical properties & polymer electrolytes	S2 only	-
89	Erol, İ.; İsmail, İ.; Demirelli, K.; Aksu, M.; Güney, B. Multifunctional PVA-CS/LaB6-MXene fiber nanocomposites: Improved thermal, electrical and biological properties. *Journal of the Indian Chemical Society*, *102*(12). 2025, doi:10.1016/j.jics.2025.102244.	Dielectric/electrical properties & polymer electrolytes	S2 only	-
90	Erol, İ.; Khamidov, G.; Acar, F. Bioengineered ZnO-Enhanced CS-based nanocomposites: Synergistic tuning of dielectric, electrical, and surface properties for Next-Gen flexible electronics. *Journal of Polymer Research*, *32*(8). 2025a, doi:10.1007/s10965-025-04487-w.	Dielectric/electrical properties & polymer electrolytes	S2 only	stretchable
91	Fang, Z.; Lou, W.; Zhang, W.; Guan, X.; He, J.; Lin, J. Modulating crystallinity and dielectric constant of chitosan film for triboelectric polarity shift and performance enhancement in triboelectric nanogenerators. *Nano Energy*, *117*. 2023, doi:10.1016/j.nanoen.2023.108923.	Dielectric/electrical properties & polymer electrolytes	S2 only	stretchable
92	Gorshkova, N.; Palamarchuk, I.; Brovko, O.; Volkov, A.; Malkov, A.; Bogdanovich, N.; Ivakhnov, A.; Chukhchin, D. Semiconductor carbon-nitrogen nanomaterials based on interpolyelectrolyte complex sodium lignosulfonate-chitosan. *Journal of Wood Chemistry and Technology*, *42*(4), 253--264. 2022, doi:10.1080/02773813.2022.2080224.	Dielectric/electrical properties & polymer electrolytes	S2 only	conductive
93	Güzel, İ.; Yetiş, A.S.; Abaci, U.; Okkay, H.; Yılmazoğlu, M. Dielectric and proton conductivity potential of sulfonated silica-enhanced chitosan/butylimidazolium tetrafluoroborate membrane electrolytes. *Colloids and Surfaces A: Physicochemical and Engineering Aspects*, *727*. 2025, doi:10.1016/j.colsurfa.2025.138254.	Dielectric/electrical properties & polymer electrolytes	S2 only	-
94	Hadi, J.M.; Aziz, S.B.; Saeed, S.R.; Brza, M.A.; Abdulwahid, R.T.; Hamsan, M.H.; Abdullah, R.M.; Kadir, M.F.Z.; Muzakir, S.K. Investigation of ion transport parameters and electrochemical performance of plasticized biocompatible chitosan-based proton conducting polymer composite electrolytes. *Membranes*, *10*(11), 1--27. 2020a, doi:10.3390/membranes10110363.	Dielectric/electrical properties & polymer electrolytes	S2 only	-
95	Hadi, J.M.; Aziz, S.B.; Abdulwahid, R.T.; Brza, M.A.; Tahir, H.B.; Hamad, S.M.; Shamsuri, N.A.; Woo, H.J.; Alias, Y.; Hamsan, M.H.; Steve, O.J.S.; Kadir, M.F.Z. Characterizing sodium-conducting biopolymer blend electrolytes with glycerol plasticizer for EDLC application. *Ionics*, *30*(4), 2409--2423. 2024, doi:10.1007/s11581-024-05412-9.	Dielectric/electrical properties & polymer electrolytes	S2 only	-
96	Hamsan, M.H.; Aziz, S.B.; Kadir, M.F.Z.; Brza, M.A.; Karim, W.O. The study of EDLC device fabricated from plasticized magnesium ion conducting chitosan based polymer electrolyte. *Polymer Testing*, *90*. 2020, doi:10.1016/j.polymertesting.2020.106714.	Dielectric/electrical properties & polymer electrolytes	S2 only	-
97	Hassan, M.M.; Bristi, A.A.; He, X.; Trifkovic, M.; Bobrov, G.; Lu, Q. Novel nanoarchitecture of 3D ion transfer channel containing nanocomposite solid	Dielectric/electrical properties &	S2 only	stretchable

Ref	Citation	Theme	Source	Emerging-topic flag
	polymer electrolyte membrane based on holey graphene oxide and chitosan biopolymer. *Chemical Engineering Journal*, *466*. 2023, doi:10.1016/j.cej.2023.143159.	polymer electrolytes		
98	Hassan, M.M.; Wang, X.-Y.; Bristi, A.A.; Yang, R.; Li, X.; Lu, Q. Composite scaffold of electrospun nano-porous cellulose acetate membrane casted with chitosan for flexible solid-state sodium-ion batteries. *Nano Energy*, *128*. 2024, doi:10.1016/j.nanoen.2024.109971.	Dielectric/electrical properties & polymer electrolytes	S2 only	stretchable
99	Hassan, M.M.; Conners, B.; Mashhadi, M.E.; Hu, J.; Li, X.; Lu, Q. Controlled Nanoarchitecture of Aligned Electrospinning Fiber Composite Electrolyte Based on Plant and Animal Biopolymers for Safe Na-Ion Batteries. *Energy and Environmental Materials*. 2026, doi:10.1002/eem2.70289.	Dielectric/electrical properties & polymer electrolytes	S2 only	stretchable
100	Hazarika, D.; Kamei, D.; Shijoh, N.; Choudhury, N.A. An Ionically Cross-Linked Chitosan Hydrogel Membrane Electrolyte for Long-Lived Electrical Double Layer Capacitors. *J. Power Sources* **2024**, *629*, 236037, doi:10.1016/j.jpowsour.2024.236037.	Dielectric/electrical properties & polymer electrolytes	Main-text	-
101	Helen, P.A.; Selvin, P.C.; Sakthivel, P. Quasi-solid-state plasticized chitosan biopolymer electrolyte with enhanced Mg2+ ion mobility for next-generation Mg ion battery. *Journal of Solid State Electrochemistry*, *28*(9), 3147--3161. 2024, doi:10.1007/s10008-024-05867-w.	Dielectric/electrical properties & polymer electrolytes	S2 only	-
102	Huang, X.-W.; Liao, S.-Y.; Liu, Y.-D.; Rao, Q.-S.; Peng, X.-K.; Min, Y.-G. Design, fabrication and application of PEO/CMC-Li \@PI hybrid polymer electrolyte membrane in all-solid-state lithium battery. *Electrochimica Acta*, *389*. 2021, doi:10.1016/j.electacta.2021.138747.	Dielectric/electrical properties & polymer electrolytes	S2 only	-
103	Humelnicu, A.-C.; Samoila, P.; Asandulesa, M.; Cojocaru, C.; Bele, A.; Marinoiu, A.T.; Sacca, A.; Harabagiu, V. Chitosan-sulfated titania composite membranes with potential applications in fuel cell: Influence of cross-linker nature. *Polymers*, *12*(5). 2020a, doi:10.3390/POLYM12051125.	Dielectric/electrical properties & polymer electrolytes	S2 only	-
104	Ismadi, A.S.N.B.; Jaafar, N.K.B.; Yusoff, M.Z.B.M. Structure and Electrical Properties of Polymer Electrolyte Based on Plasticized Chitosan Grafted Polymethyl Methacrylate (Ch-g-PMMA)-Magnesium Triflate for Electrochemical Double Layer Capacitor. *Trends in Sciences*, *21*(3). 2024, doi:10.48048/tis.2024.7317.	Dielectric/electrical properties & polymer electrolytes	S2 only	-
105	Kang, L.-L.; Zuo, S.-W.; Han, B.-X.; Li, G. Comparative study on proton conductivity of a polypyridinyl multicarboxylate-based hydrogen-bonded organic framework and related chitosan composite membrane. *New Journal of Chemistry*, *47*(3), 1299--1307. 2022, doi:10.1039/d2nj04779a.	Dielectric/electrical properties & polymer electrolytes	S2 only	-
106	Kaur, D.; Panwar, R.S.; Kumar, N.; Madhu, C.; Jindal, S. Enhanced dielectric and ferroelectric characteristics of CS-ZnO and CS-TZO nanocomposite thin films for high energy storage density. *Journal of Materials Science: Materials in Electronics*, *36*(16). 2025a, doi:10.1007/s10854-025-15012-6.	Dielectric/electrical properties & polymer electrolytes	S2 only	-
107	Leones, R.; Sentanin, F.; Nunes, S.C.; Esperança, J.M.S.S.; Gonçalves, M.C.; Pawlicka, A.; Bermudez, V.D.Z.; Silva, M.M. Effect of the alkyl chain length of the ionic liquid anion on polymer electrolytes properties. *Electrochimica Acta*, *184*, 171--178. 2015, doi:10.1016/j.electacta.2015.09.163.	Dielectric/electrical properties & polymer electrolytes	S2 only	-
108	Majumdar, S.; Sen, P.; Ray, R. High-performance graphene oxide-grafted chitosan-starch solid biopolymer electrolytes for flexible hybrid supercapacitors. *Journal of Solid State Electrochemistry*, *26*(2), 527--547. 2022, doi:10.1007/s10008-021-05093-8.	Dielectric/electrical properties & polymer electrolytes	S2 only	-
109	Meera, K.; Ramesan, M.T. Performance of Boehmite Nanoparticles Reinforced Carboxymethyl Chitosan/Polyvinyl Alcohol Blend Nanocomposites Tailored Through Green Synthesis. *Journal of Polymers and the Environment*, *31*(2), 447--460. 2023b, doi:10.1007/s10924-022-02649-1.	Dielectric/electrical properties & polymer electrolytes	S2 only	-
110	Meera, K.; Ramesan, M.T. Tailoring the performance of boehmite nanoparticles reinforced carboxymethyl chitosan/cashew gum blend nanocomposites via green synthesis. *Polymer*, *268*. 2023c, doi:10.1016/j.polymer.2023.125706.	Dielectric/electrical properties & polymer electrolytes	S2 only	-
111	Mısırlıoğlu, B.S.; Berber, H.; Gül, E.; Oladipo, A.A.; Gazi, M. Thin and flexible $Mg_{0.7}Ni_{0.3}Fe_2O_4$ nanoparticle-reinforced chitosan/polyvinyl alcohol bionanocomposite films with tunable dielectric properties for energy storage devices. *International Journal of Biological Macromolecules*, *339*. 2026, doi:10.1016/j.ijbiomac.2025.149980.	Dielectric/electrical properties & polymer electrolytes	S2 only	-

Ref	Citation	Theme	Source	Emerging-topic flag
112	Mohamed, A.S.; Asnawi, A.S.F.M.; Shukur, M.F.; Matmin, J.; Kadir, M.F.Z.; Yusof, Y.M. The Development of Chitosan-Maltodextrin Polymer Electrolyte with the Addition of Ionic Liquid for Electrochemical Double Layer Capacitor (EDLC) Application. *International Journal of Electrochemical Science*, *17*. 2022, doi:10.20964/2022.03.30.	Dielectric/electrical properties & polymer electrolytes	S2 only	-
114	Mohammed, S.S.; Hamid, D.A.; Salih, I.L.; Aziz, S.B.; Babakr, K.A.; Mahmood, P.H.; Ibrahim, P.A.; Qader, I.N.; Muhammed, A.W.; Rasul, H.H.; Aspoukeh, P.; Hamad, S.M.; Khojasteh, H. Glycerol-plasticized chitosan--sorbitol/NaNO3electrolytes reinforced with TiO2nanofillers: A structural and ionic study. *Journal of Elastomers and Plastics*, *58*(1), 46--72. 2026, doi:10.1177/00952443251378992.	Dielectric/electrical properties & polymer electrolytes	S2 only	-
116	Mumtazah, Z.; Khotimah, H.; Priyangga, A.; Nugraha, R.E.; Qonitatillah, H.; Safitri, S.D.; Palupi, B.; Putri, D.K.Y. Performance Evaluation of Sulfonated Chitosan-TEOS Membranes: Implications for Direct Methanol Fuel Cell Applications. *Journal of Membrane Science and Research*, *11*(1). 2025, doi:10.22079/jmsr.2024.2045005.1683.	Dielectric/electrical properties & polymer electrolytes	S2 only	-
117	Na, R.; Huo, G.; Zhang, S.; Huo, P.; Du, Y.; Luan, J.; Zhu, K.; Wang, G. A novel poly(ethylene glycol)-grafted poly(arylene ether ketone) blend micro-porous polymer electrolyte for solid-state electric double layer capacitors formed by incorporating a chitosan-based LiClO4 gel electrolyte. *Journal of Materials Chemistry A*, *4*(46), 18116--18127. 2016, doi:10.1039/c6ta07846j.	Dielectric/electrical properties & polymer electrolytes	S2 only	-
118	Naffati, N.; Fernandes, M.; de Zea Bermudez, V.; Nsib, M.F.; Arfaoui, Y.; Houas, A.; Faria, J.L.; Silva, C.G.; Silva, M.M. Chitosan-based electrolytes containing carbon nanotube-titanium dioxide for energy conversion devices applications. *Iranian Polymer Journal (English Edition)*, *31*(10), 1197--1208. 2022, doi:10.1007/s13726-022-01069-1.	Dielectric/electrical properties & polymer electrolytes	S2 only	-
119	Pongampai, S.; Charoonsuk, T.; Pinpru, N.; Muanghlua, R.; Vittayakorn, W.; Vittayakorn, N. High Performance Flexible Tribo/Piezoelectric Nanogenerators based on BaTiO3/Chitosan Composites. *Integrated Ferroelectrics*, *223*(1), 137--151. 2021, doi:10.1080/10584587.2021.1964293.	Dielectric/electrical properties & polymer electrolytes	S2 only	stretchable
120	Prajapati, R.K.; Kumar, H.; Saroj, A.L. Formation of silver particles in \[PVA:CS:PEG\]-AgNO3 based biopolymer electrolyte membranes: Structural and electric transport properties study. *Physica B: Condensed Matter*, *662*. 2023, doi:10.1016/j.physb.2023.414962.	Dielectric/electrical properties & polymer electrolytes	S2 only	-
121	Prajapati, R.K.; Rawat, P.; Kumar, H.; Saroj, A.L. Chitosan-PVA-Mg(NO3)2 based biopolymer blend electrolyte membranes for EDLC applications: Structural, thermal and electrical transport properties study. *Materials Science and Engineering: B*, *317*. 2025, doi:10.1016/j.mseb.2025.118200.	Dielectric/electrical properties & polymer electrolytes	S2 only	-
122	Prajapati, R.K.; Meena, G.K.; Kumar, H.; Saroj, A.L. Chitosan-PVA-AgNO3-Ionic Liquid Based Solid Electrolyte Membranes for Supercapacitor Applications: Structural, Thermal and Ion Transport Studies. *Journal of Polymers and the Environment*, *34*(4). 2026, doi:10.1007/s10924-026-03818-2.	Dielectric/electrical properties & polymer electrolytes	S2 only	-
123	Rahman, N.A.; Hanifah, S.A.; Mobarak, N.N.; Su'ait, M.S.; Ahmad, A.; Shyuan, L.K.; Khoon, L.T. Synthesis and characterizations of o-nitrochitosan based biopolymer electrolyte for electrochemical devices. *PLoS ONE*, *14*(2). 2019, doi:10.1371/journal.pone.0212066.	Dielectric/electrical properties & polymer electrolytes	S2 only	-
124	Ramesan, M.T.; Kalladi, A.J.; Labeeba Abdulla, A.C.; Sunojkumar, P.; Bahuleyan, B.K. Development of biopolymer composite films based on chicory extract reinforced polyvinyl alcohol/chitosan blend via green approach for flexible optoelectrical devices. *Journal of Applied Polymer Science*, *141*(13). 2024, doi:10.1002/app.55154.	Dielectric/electrical properties & polymer electrolytes	S2 only	stretchable
125	Rasul, H.H.; Qader, I.N.; Mohammed, S.S.; Aziz, S.B.; Salih, I.L.; Hamid, D.A.; Ibrahim, P.A.; Muhammed, A.W.; Babakr, K.A.; Ali, B.T.; Aspoukeh, P.; Khojasteh, H.; Hamad, S.M.; Mahmood, P.H. Glycerol Plasticization Boosts Ionic Conductivity in NH4SCN/ZnO-Doped Chitosan--Dextran Electrolytes. *Journal of Electronic Materials*, *55*(3), 2702--2727. 2026a, doi:10.1007/s11664-025-12633-9.	Dielectric/electrical properties & polymer electrolytes	S2 only	-
126	Rawat, P.; Meena, G.K.; Saroj, A.L. Imidazolium cation ionic liquid-chitosan/PVP based plasticized nanocomposite biopolymer electrolyte membranes for dye-sensitized solar cell and supercapacitor applications.	Dielectric/electrical properties &	S2 only	-

Ref	Citation	Theme	Source	Emerging-topic flag
	Journal of Physics and Chemistry of Solids, *209*. 2026, doi:10.1016/j.jpcs.2025.113318.	polymer electrolytes		
127	Rizal, A.A.M.; Hassan, O.H.; Jaafar, N.K.; Mustaffa, M.; Ali, M.T.M.; Lepit, A.; Aini, N.A. Influence of Sulfonated Chitosan on Conductivity of Sulfonated Polyether Ether Ketone (SPEEK) at Room Temperature. *Energy Engineering: Journal of the Association of Energy Engineering*, *123*(1), 1--2. 2026, doi:10.32604/ee.2025.071726.	Dielectric/electrical properties & polymer electrolytes	S2 only	-
128	Ronnasi, B.; Tousignant, M.N.; Lessard, B.H. Chitosan based dielectrics for use in single walled carbon nanotube-based thin film transistors. *Journal of Materials Chemistry C*, *11*(9), 3197--3205. 2023, doi:10.1039/d2tc04458g.	Dielectric/electrical properties & polymer electrolytes	S2 only	-
132	Singh, R.; Lin, Y.-T.; Ko, F.-H. Aqueous Solution-Processable, Flexible Thin-Film Transistors Based on Crosslinked Chitosan Dielectric Thin Films. *Macromol. Mater. Eng.* **2018**, *303*, doi:10.1002/mame.201700468.	Dielectric/electrical properties & polymer electrolytes	Main-text	stretchable
133	Swaghatha, A.I.A.K.; Cindrella, L. Assessment of proton conductivity, dielectric relaxation and other physicochemical properties of LTA zeolite blended chitosan composites for membrane applications. *Reactive and Functional Polymers*, *170*. 2022, doi:10.1016/j.reactfunctpolym.2021.105116.	Dielectric/electrical properties & polymer electrolytes	S2 only	-
134	Tiwari, R.; Parwati, K.; Verma, D.K.; Kumar, D.; Yadav, S.; Rai, R.; Kumar, K.; Adhikary, P.; Krishnamoorthi, S. Ionic liquid supported chitosan-g-SPA as a biopolymer-based single ion conducting solid polymer electrolyte for energy storage devices. *International Journal of Biological Macromolecules*, *280*. 2024, doi:10.1016/j.ijbiomac.2024.135872.	Dielectric/electrical properties & polymer electrolytes	S2 only	-
135	Wolf, M.H.; Izaguirre, N.; Pascual-José, B.; Teruel-Juanes, R.; Labidi, J.; Ribes-Greus, A. Dielectric characterisation of chitosan-based composite membranes containing fractionated kraft and organosolv lignin. *Reactive and Functional Polymers*, *196*. 2024, doi:10.1016/j.reactfunctpolym.2024.105833.	Dielectric/electrical properties & polymer electrolytes	S2 only	-
136	Wolf, M.H.; Izaguirre, N.; Labidi, J.; Ribes-Greus, A. Dielectric Characterization of Protonated Chitosan-Lignin Biocomposite Membranes: Influence of Chitosan and Lignin Types. *Biomacromolecules*, *26*(9), 6140--6151. 2025a, doi:10.1021/acs.biomac.5c01090.	Dielectric/electrical properties & polymer electrolytes	S2 only	-
137	Yesilyurt, E.I.; Pionteck, J.; Keskinen, J.; Kattainen, A.; Punkari, T.; Simon, F.; Mäntysalo, M.; Voit, B. Screen printable PANI/carbide-derived carbon supercapacitor electrode ink with chitosan binder. *Flexible and Printed Electronics*, *8*(4). 2023a, doi:10.1088/2058-8585/ad0ebd.	Dielectric/electrical properties & polymer electrolytes	S2 only	conductive
138	Yılmazoğlu, M.; Okkay, H.; Abaci, U.; Tekay, E.; Coban, O.; Veziroğlu, S.; Yahsi, A.Y.; Tav, C.; Yahsi, U. Graphene oxide-induced free volume effects in proton conductive chitosan/polyvinyl alcohol (CS/PVA)-based composite electrolytes: A positron annihilation lifetime study. *Journal of Materials Science: Materials in Electronics*, *36*(30). 2025, doi:10.1007/s10854-025-16027-9.	Dielectric/electrical properties & polymer electrolytes	S2 only	-
139	Yu, M.; Ji, X.; Ran, F. Chemically building interpenetrating polymeric networks of Bi-crosslinked hydrogel macromolecules for membrane supercapacitors. *Carbohydrate Polymers*, *255*. 2021, doi:10.1016/j.carbpol.2020.117346.	Dielectric/electrical properties & polymer electrolytes	S2 only	stretchable
141	Zhou, L.; Liu, S.; Li, W.; Song, H.; Du, L.; Cui, Z. Highly conductive Poly(ε-caprolactone) and chitosan based polymer electrolyte for lithium metal battery. *Journal of Power Sources*, *553*. 2023, doi:10.1016/j.jpowsour.2022.232271.	Dielectric/electrical properties & polymer electrolytes	S2 only	-
142	Alaei, I.; Al-Bat'hi, S.A.M.; Sopyan, I. Fabrication and characterization of poly (ethylene oxide) for photo-electronic devices. *ARPN Journal of Engineering and Applied Sciences*, *10*(22), 17239--17241. 2015.	Optoelectronic & band-gap engineering	S2 only	-
143	Alsobai, N.A.; Elamin, N.Y.; Albqmi, M.; Basfer, N.M.; Elamin, M.R.; Farea, M.O.; Abdelrazek, E.M.; Rajeh, A. Studies on the Role of Bi2O3 NPs in Imparting Unique Optical, Dielectric, and Conductivity Properties to Nanocomposites for Energy Storage Devices. *Polymer Engineering and Science*, *66*(4), 3047--3057. 2026a, doi:10.1002/pen.70424.	Optoelectronic & band-gap engineering	S2 only	-

Ref	Citation	Theme	Source	Emerging-topic flag
144	Aziz, S.B.; Rasheed, M.A.; Abidin, Z.H.Z. Optical and Electrical Characteristics of Silver Ion Conducting Nanocomposite Solid Polymer Electrolytes Based on Chitosan. *Journal of Electronic Materials*, *46*(10), 6119--6130. 2017, doi:10.1007/s11664-017-5515-8.	Optoelectronic & band-gap engineering	S2 only	-
145	El Sayed, A.M.; Abdel Maksoud, M.I.A.; Kassem, S.M.; Awed, A.S. Synthesis, structural, optical, and dielectric properties of CuWO4/PVP/Cs bio-nanocomposites for some industrial applications. *Journal of Materials Science: Materials in Electronics*, *34*(24). 2023, doi:10.1007/s10854-023-11118-x.	Optoelectronic & band-gap engineering	S2 only	-
146	Erol, İ.; Khamidov, G.; Mutlu, T. Surface-Enhanced Optical and Electrical Performance of CS-Based Nanocomposites Containing Ag-ZnO: A Structural and Morphological Perspective. *Plasmonics*, *21*(1), 1465--1489. 2026, doi:10.1007/s11468-025-03304-w.	Optoelectronic & band-gap engineering	S2 only	-
147	Leones, R.; Reis, P.M.; Sabadini, R.C.; Ravaro, L.P.; Silva, I.D.A.; de Camargo, A.S.S.; Donoso, J.P.; Magon, C.J.; Esperança, J.M.S.S.; Pawlicka, A.; Silva, M.M. A luminescent europium ionic liquid to improve the performance of chitosan polymer electrolytes. *Electrochimica Acta*, *240*, 474--485. 2017a, doi:10.1016/j.electacta.2017.04.042.	Optoelectronic & band-gap engineering	S2 only	-
148	Lokesh Kumar, S.; Servesh, A.; Sunitha, V.R.; Prasad, J.R.; Tabassum, S.; Ramaraj, S.G.; Kumar, N.; Govindaraju, S. Modified eco-friendly and biodegradable chitosan-based sustainable semiconducting thin films. *Polymers for Advanced Technologies*, *35*(6). 2024a, doi:10.1002/pat.6470.	Optoelectronic & band-gap engineering	S2 only	-
149	Morsi, M.A.; Al-Sulami, A.I.; Al Sulami, F.M.H.; Farea, M.O.; Alqarni, M.A.; Alhazime, A.A.; Rajeh, A. Preparation, structural characterization, optical, photoluminescence, AC electrical conductivity and broadband dielectric properties of WO3 reinforced PEG/CS blend for futuristic optoelectronic and energy storage devices. *Results in Physics*, *59*. 2024, doi:10.1016/j.rinp.2024.107582.	Optoelectronic & band-gap engineering	S2 only	-
150	Ramesan, M.T.; Labeeba Abdulla, A.C.; Kalladi, A.J.; Sunojkumar, P. Biopolymer blend composite films based on polyvinyl alcohol/ chitosan/ grape seed extract via green approach for flexible optoelectronic devices. *Journal of Thermoplastic Composite Materials*, *37*(12), 3697--3714. 2024, doi:10.1177/08927057241238986.	Optoelectronic & band-gap engineering	S2 only	stretchable
151	Ramesan, M.T.; Kalladi, A.J.; Mathew, J.; Meera, K.; Sankar, S.; Verma, M. Reinforcement of Asparagus racemosus as a bio-filler agent in polyvinyl alcohol/chitosan blend for flexible electronic devices using a green approach. *Research on Chemical Intermediates*, *50*(1), 465--483. 2024a, doi:10.1007/s11164-023-05163-8.	Optoelectronic & band-gap engineering	S2 only	stretchable
152	Saadi, M.; Erouel, M.; Tall, A.; Haba, S.; Bouguila, N.; Jaffrezic-Renault, N.; Khirouni, K. Morphological, Optical, and Dielectric Properties of Chitosan Biopolymer Thin Films Synthesized by Spray Pyrolysis. *ACS Omega*, *10*(40), 46384--46392. 2025a, doi:10.1021/acsomega.5c00793.	Optoelectronic & band-gap engineering	S2 only	stretchable
153	Sankar, S.; Krishnaraj, T.P.; Sunojkumar, P.; Ramesan, M.T. Chitosan-functionalized poly(thiophene-co-pyrrole) nanocomposites: Promising materials for optoelectronics and antibacterial surfaces. *Journal of Molecular Liquids*, *437*. 2025, doi:10.1016/j.molliq.2025.128319.	Optoelectronic & band-gap engineering	S2 only	conductive
154	Viorica, G.P.; Musat, V.; Pimentel, A.; Calmeiro, T.R.; Carlos, E.; Baroiu, L.; Martins, R.; Fortunato, E. Hybrid (Ag)ZnO/Cs/PMMA nanocomposite thin films. *Journal of Alloys and Compounds*, *803*, 922--933. 2019a, doi:10.1016/j.jallcom.2019.06.373.	Optoelectronic & band-gap engineering	S2 only	-
155	Zidani, J.; Hassine, K.; Zannen, M.; Zeinert, A.; Da Costa, A.; Ferri, A.; Belhadi, J.; Majdoub, M.; El Marssi, M.; Lahmar, A. Synthesis, Structural, Optical, and Electrical Characterization of Biochitosan/Na0.5Bi0.5TiO3 Composite Thin-Film Materials. *Micromachines*, *14*(10). 2023a, doi:10.3390/mi14101841.	Optoelectronic & band-gap engineering	S2 only	-
157	Bansod, N.H.; Chaudhari, G.N.; Bodade, A.B.; Kharat, P.B. Sol-Gel Derived Cobalt-Doped CeO2 Nanocomposite on Gold Electrode for Glucose Electrochemical Sensing. *Asian Journal of Chemistry*, *34*(5), 1255--1262. 2022, doi:10.14233/ajchem.2022.23684.	Electrochemical, sensing & smart devices	S2 only	-
160	Chen, S.; Chen, X.; Xia, T.; Ma, Q. A novel electrochemiluminescence sensor for the detection of nitroaniline based on the nitrogen-doped graphene	Electrochemical, sensing & smart devices	S2 only	-

Ref	Citation	Theme	Source	Emerging-topic flag
	quantum dots. *Biosensors and Bioelectronics*, *85*, 903--908. 2016, doi:10.1016/j.bios.2016.06.010.			
162	Dou, W.; Tan, Y. Junctionless Dual In-Plane-Gate Thin-Film Transistors with and Logic Function on Paper Substrates. *ACS Omega*, *4*(25), 21417--21420. 2019, doi:10.1021/acsomega.9b03118.	Electrochemical, sensing & smart devices	S2 only	-
163	Du, B.-W.; Hu, S.-Y.; Singh, R.; Tsai, T.-T.; Lin, C.-C.; Ko, F.-H. Eco-friendly and biodegradable biopolymer chitosan/Y2O3 composite materials in flexible organic thin-film transistors. *Materials*, *10*(9). 2017a, doi:10.3390/ma10091026.	Electrochemical, sensing & smart devices	S2 only	stretchable
164	Gan, X.; Dou, W.; Hou, W.; Yuan, X.; Lei, L.; Zhou, Y.; Yang, J.; Chen, D.; Zhou, W.; Tang, D. Low-Voltage Solution-Processed Zinc-Doped CuI Thin Film Transistors with NOR Logic and Artificial Synaptic Function. *Nanomaterials*, *13*(16). 2023, doi:10.3390/nano13162345.	Electrochemical, sensing & smart devices	S2 only	-
165	Garcia-Torres, J.; Crean, C. Multilayered Flexible Fibers with High Performance for Wearable Supercapacitor Applications. *Advanced Sustainable Systems*, *2*(2). 2018, doi:10.1002/adsu.201700143.	Electrochemical, sensing & smart devices	S2 only	stretchable
166	Jakobczyk, P.; Kaczmarzyk, I.; Bogdanowicz, R. Few-Layer Black Phosphorus/Chitosan Nanocomposite Electrodes via Controlled Electrodeposition for Enhanced Electrochemical Kinetic Performance. *Journal of Physical Chemistry C*, *128*(17), 7087--7095. 2024, doi:10.1021/acs.jpcc.3c08361.	Electrochemical, sensing & smart devices	S2 only	-
167	Jalalvand, A.R.; Haseli, A.; Farzadfar, F.; Goicoechea, H.C. Fabrication of a novel biosensor for biosensing of bisphenol A and detection of its damage to DNA. *Talanta*, *201*, 350--357. 2019, doi:10.1016/j.talanta.2019.04.037.	Electrochemical, sensing & smart devices	S2 only	-
168	Jiang, W.; Peng, C.; Yuan, Y.; Yang, S.; Li, X. Bulk preparation of transparent IGZO TFTs with CS gate dielectric as glucose sensor on a pilot line. *Journal of Materials Science: Materials in Electronics*, *31*(2), 1547--1553. 2020, doi:10.1007/s10854-019-02671-5.	Electrochemical, sensing & smart devices	S2 only	-
169	Kang, Z.; Jiao, K.; Yu, C.; Dong, J.; Peng, R.; Hu, Z.; Jiao, S. Direct electrochemistry and bioelectrocatalysis of glucose oxidase in CS/CNC film and its application in glucose biosensing and biofuel cells. *RSC Advances*, *7*(8), 4572--4579. 2017, doi:10.1039/C6RA26636C.	Electrochemical, sensing & smart devices	S2 only	-
171	Ke, S.; He, Y.; Zhu, L.; Jiang, Z.; Mao, H.; Zhu, Y.; Wan, C.; Wan, Q. Indium-Gallium-Zinc-Oxide Based Photoelectric Neuromorphic Transistors for Modulable Photoexcited Corneal Nociceptor Emulation. *Advanced Electronic Materials*, *7*(11). 2021, doi:10.1002/aelm.202100487.	Electrochemical, sensing & smart devices	S2 only	-
172	Krishna, R.; Campiña, J.M.; Fernandes, P.M.V.; Ventura, J.; Titus, E.; Silva, A.F. Reduced graphene oxide-nickel nanoparticles/biopolymer composite films for the sub-millimolar detection of glucose. *Analyst*, *141*(13), 4151--4161. 2016, doi:10.1039/c6an00475j.	Electrochemical, sensing & smart devices	S2 only	-
173	Li, X.; Jiang, L.; Yan, M.; Bi, H.; Wang, Q. Highly Stretchable, Tough and Conductive Chitin Nanofiber Composite Hydrogel as a Wearable Sensor. *Int. J. Biol. Macromol.* **2023**, *242*, doi:10.1016/j.ijbiomac.2023.124780.	Electrochemical, sensing & smart devices	Main-text	stretchable
175	Liu, R.; Zhu, L.Q.; Wang, W.; Hui, X.; Liu, Z.P.; Wan, Q. Biodegradable oxide synaptic transistors gated by a biopolymer electrolyte. *Journal of Materials Chemistry C*, *4*(33), 7744--7750. 2016, doi:10.1039/c6tc02693a.	Electrochemical, sensing & smart devices	S2 only	-
176	Liu, M.; Huang, G.; Feng, P.; Guo, Q.; Shao, F.; Tian, Z.; Li, G.; Wan, Q.; Mei, Y. Artificial neuron synapse transistor based on silicon nanomembrane on plastic substrate. *Journal of Semiconductors*, *38*(6). 2017, doi:10.1088/1674-4926/38/6/064006.	Electrochemical, sensing & smart devices	S2 only	-
177	Lu, G.; Liu, Y.; Lin, F.; Gen, K.; Wu, W.; Yao, R. Realization of artificial synapse and inverter based on oxide electric-double-layer transistor gated by a chitosan biopolymer electrolyte. *Semiconductor Science and Technology*, *35*(7). 2020, doi:10.1088/1361-6641/ab883e.	Electrochemical, sensing & smart devices	S2 only	-
178	Ma, C.; He, Y.; Zeng, L.; Liu, M. Surface Modification of Chitin Nanocrystals Using Conducting Polymer for Triboelectric Nanogenerator. *Nano Energy* **2025**, *135*, 110660, doi:10.1016/j.nanoen.2025.110660.	Electrochemical, sensing & smart devices	Main-text	conductive

Ref	Citation	Theme	Source	Emerging-topic flag
179	Maizal Hairi, N.I.I.; Md Ralib, A.A.; Ahmad, F.B.; bt Mat Hattar, M.A. Synthesis and Characterization of Different Graphite/Chitosan Ink Ratio Composition towards Flexible Strain Sensor Performance. *J Mater Sci Mater Electron* **2022**, *33*, 15574--15585, doi:10.1007/s10854-022-08463-8.	Electrochemical, sensing & smart devices	Main-text	stretchable
180	Majumdar, S.; Antony, A.M.; Kulkarni, G.U. A 7.5 V window dual-functional planar micro-device based on a biopolymer ionogel electrolyte for charge storage and neuromorphic computing. *Journal of Materials Chemistry A*, *13*(17), 12349--12363. 2025, doi:10.1039/d5ta00490j.	Electrochemical, sensing & smart devices	S2 only	-
181	Min, S.-Y.; Cho, W.-J. CMOS-compatible synaptic transistor gated by chitosan electrolyte-Ta2O5 hybrid electric double layer. *Scientific Reports*, *10*(1). 2020, doi:10.1038/s41598-020-72684-2.	Electrochemical, sensing & smart devices	S2 only	-
182	Min, J.-G.; Cho, W.-J. Sol-gel composites-based flexible and transparent amorphous indium gallium zinc oxide thin-film synaptic transistors for wearable intelligent electronics. *Molecules*, *26*(23). 2021, doi:10.3390/molecules26237233.	Electrochemical, sensing & smart devices	S2 only	stretchable
183	Pan, Q.; Wu, Q.; Sun, Q.; Zhou, X.; Cheng, L.; Zhang, S.; Yuan, Y.; Zhang, Z.; Ma, J.; Zhang, Y.; Zhu, B. Biomolecule-friendly conducting PEDOT interface for long-term bioelectronic devices. *Sensors and Actuators B: Chemical*, *373*. 2022, doi:10.1016/j.snb.2022.132703.	Electrochemical, sensing & smart devices	S2 only	conductive
184	Park, K.-W.; Cho, W.-J. Time-Dependent Sensitivity Tunable pH Sensors Based on the Organic-Inorganic Hybrid Electric-Double-Layer Transistor. *International Journal of Molecular Sciences*, *23*(18). 2022, doi:10.3390/ijms231810842.	Electrochemical, sensing & smart devices	S2 only	-
185	Plekhanova, Y.; Tarasov, S.; Kolesov, V.; Kuznetsova, I.; Signore, M.; Quaranta, F.; Reshetilov, A. Effects of polymer matrices and carbon nanotubes on the generation of electric energy in a microbial fuel cell. *Membranes*, *8*(4). 2018, doi:10.3390/membranes8040099.	Electrochemical, sensing & smart devices	S2 only	-
186	Sahu, P.K.; Gangwar, R.; Ramesh, A.; Rao, K.T.; Vanjari, S.R.K.; Subrahmanyam, C. Green-Synthesized Amino Carbons for Impedimetric Biosensing of E. coli O157:H7. *ACS Infectious Diseases*, *10*(5), 1644--1653. 2024, doi:10.1021/acsinfecdis.3c00721.	Electrochemical, sensing & smart devices	S2 only	-
187	Salikhov, R.; Zilberg, R.; Mullagaliev, I.; Salikhov, T.; Teres, Y.; Bulysheva, E.; Ostaltsova, A. Composite and Nanocomposite Thin-Film Structures Based on Chitosan Succinamide. *Curr. Nanomedicine* **2025**, *15*, 70--79, doi:10.2174/0124681873279865231126034248.	Electrochemical, sensing & smart devices	Main-text	conductive
188	Shafiei, F.; Saberi, R.S.; Mehrgardi, M.A. A label-free electrochemical aptasensor for breast cancer cell detection based on a reduced graphene oxide-chitosan-gold nanoparticle composite. *Bioelectrochemistry*, *140*. 2021, doi:10.1016/j.bioelechem.2021.107807.	Electrochemical, sensing & smart devices	S2 only	-
189	Shitanda, I.; Samori, T.; Satake, M.; Loew, N.; Motosuke, M.; Mukaimoto, T.; Yanagita, S.; Suzuki, T.; Mikawa, T.; Tsujimura, S.; Watanabe, H.; Itagaki, M. Wearable Self-Powered Biosensor for Continuous Lactate Monitoring in Sweat. *ChemElectroChem*, *12*(20). 2025, doi:10.1002/celc.202500222.	Electrochemical, sensing & smart devices	S2 only	stretchable
190	Sousa, C.P.; de Oliveira, R.C.; Freire, T.M.; Fechine, P.B.A.; Salvador, M.A.; Homem-de-Mello, P.; Morais, S.; de Lima-Neto, P.; Correia, A.N. Chlorhexidine digluconate on chitosan-magnetic iron oxide nanoparticles modified electrode: Electroanalysis and mechanistic insights by computational simulations. *Sensors and Actuators, B: Chemical*, *240*, 417--425. 2017, doi:10.1016/j.snb.2016.08.181.	Electrochemical, sensing & smart devices	S2 only	-
191	Wu, G.; Xiao, H. Paste-type thin-film transistors based on self-supported chitosan membranes. *RSC Advances*, *5*(127), 105084--105089. 2015, doi:10.1039/c5ra22065c.	Electrochemical, sensing & smart devices	S2 only	stretchable
193	Ye, Z.; Meng, Q.; Yu, H.; Shi, S.; Wang, Y.; Lan, Z.; Liao, J.; Sun, Q.; Shen, X. Cellulose/chitosan film based triboelectric-piezoelectric coupled nanogenerator for wearable mechanosensing and energy harvesting. *International Journal of Biological Macromolecules*, *315*. 2025a, doi:10.1016/j.ijbiomac.2025.144647.	Electrochemical, sensing & smart devices	S2 only	stretchable
195	Zhang, X.; Zhang, H.; Lv, X.; Xie, T.; Chen, J.; Fang, D.; Yi, S. One-Step Ionic Liquid-Assisted Stabilization and Dispersion: Exfoliated Graphene and Its Applications in Stimuli-Responsive Conductive Hydrogels Based on	Electrochemical, sensing & smart devices	Main-text	stretchable

Ref	Citation	Theme	Source	Emerging-topic flag
	Chitosan. *Int. J. Biol. Macromol.* **2024**, *271*, doi:10.1016/j.ijbiomac.2024.132699.			
196	Zhang, J.; Yang, J.; Zhou, R.; Liu, H.; Chang, Y.; Babichuk, I.S. A Polymer Gel-Gated Flexible Transistor Based on Chitosan Physically Cross-Linked Films for Hydrogen Sulfide Gas Sensing. *Sens. Actuators A Phys.* **2025**, *390*, 116593, doi:10.1016/j.sna.2025.116593.	Electrochemical, sensing & smart devices	Main-text	stretchable
197	Zhou, Q.; Guo, M.; Wu, S.; Fornara, D.; Sarkar, B.; Sun, L.; Wang, H. Electrochemical sensor based on corncob biochar layer supported chitosan-MIPs for determination of dibutyl phthalate (DBP). *Journal of Electroanalytical Chemistry*, *897*. 2021, doi:10.1016/j.jelechem.2021.115549.	Electrochemical, sensing & smart devices	S2 only	-
198	Zilberg, R.; Salikhov, R.; Mullagaliev, I.; Teres, Y.; Bulysheva, E.; Salikhov, T.; Ostaltsova, A.; Vakulin, I. Chitosan-Based Polyelectrolyte Complex in Combination with Allotropic Forms of Carbon as a Basis of Thin-Film Organic Electronics. *Chim. Techno Acta* **2024**, *11*, doi:10.15826/chimtech.2024.11.3.02.	Electrochemical, sensing & smart devices	Main-text	conductive
199	Žutautas, V.; Jelinskas, T.; Pauliukaite, R. A novel sensor for electrochemical pH monitoring based on polyfolate. *Journal of Electroanalytical Chemistry*, *921*. 2022, doi:10.1016/j.jelechem.2022.116668.	Electrochemical, sensing & smart devices	S2 only	conductive
200	de, S.; Mohanty, S.; Nayak, S.K. Nano-CeO2 decorated graphene based chitosan nanocomposites as enzymatic biosensing platform: Fabrication and cellular biocompatibility assessment. *Bioprocess and Biosystems Engineering*, *38*(9), 1671--1683. 2015, doi:10.1007/s00449-015-1408-5.	Electrochemical, sensing & smart devices	S2 only	-

Note: Themes correspond to the four thematic groups used in the main text: (A) structural engineering and processing routes; (B) dielectric, electrical, and impedance properties / polymer electrolytes; (C) optoelectronic and band-gap engineering; (D) electrochemical, impedance-sensing, and smart-device applications. The "emerging-topic flag" marks studies specifically relevant to intrinsically conductive chitosan or stretchable/wearable chitosan-based electronics, corresponding to the under-represented directions discussed in Section 3.9. Self-healing chitosan-based electronic skin was searched for specifically but is not represented among the records retrieved by the present search strategy; this is noted in Section 3.9 as an identified gap rather than a covered theme.
